# Stress-related exposures amplify the effects of genetic susceptibility on depression and anxiety

**DOI:** 10.1038/s41398-023-02327-3

**Published:** 2023-01-30

**Authors:** Rujia Wang, Lifelines Cohort Study, Catharina A. Hartman, Harold Snieder

**Affiliations:** 1 Department of Epidemiology, University of Groningen, University Medical Center Groningen, Groningen, Netherlands; 2 Department of Psychiatry, University of Groningen, University Medical Center Groningen, Groningen, Netherlands

**Keywords:** Depression, Genetics

## Abstract

It is unclear whether and to what extent stress-related exposures moderate the effects of polygenic risk scores (PRSs) on depression and anxiety. We aimed to examine such moderation effects for a variety of stress-related exposures on depression and anxiety. We included 41,810 participants with both genome-wide genetic data and measurements of depression and anxiety in the Lifelines Cohort Study. Current depression and anxiety were measured by the MINI International Neuropsychiatric Interview. Stress-related exposures included long-term difficulties, stressful life events, reduced social support, childhood trauma, and loneliness, which were measured by self-report questionnaires. PRSs were calculated based on recent large genome-wide association studies for depression and anxiety. We used linear mixed models adjusting for family relationships to estimate the interactions between PRSs and stress-related exposures. Nine of the ten investigated interactions between the five stress-related exposures and the two PRSs for depression and anxiety were significant (*Ps* < 0.001). Reduced social support, and higher exposure to long-term difficulties, stressful life events, and loneliness amplified the genetic effects on both depression and anxiety. As for childhood trauma exposure, its interaction with the PRS was significant for depression (*P* = 1.78 × 10^–05^) but not for anxiety (*P* = 0.32). Higher levels of stress-related exposures significantly amplify the effects of genetic susceptibility on depression and anxiety. With a large sample size and a comprehensive set of stress-related exposures, our study provides powerful evidence on the presence of polygenic risk-by-environment interactions in relation to depression and anxiety.

## Introduction

Depression and anxiety are common diseases worldwide, with lifetime prevalences of 14.6% for major depressive disorder (MDD) [1], and up to 33.7% for all anxiety disorders [2]. Depression and anxiety may have severe consequences, such as reduced social functioning and workability [3], low health-related quality of life [4], and elevated suicide rates [5]. These disorders aggregate within families and are moderately heritable [6], with twin heritabilities of 0.37 for MDD [7], and 0.32 for generalized anxiety disorder (GAD) [8]. Recently, two genome-wide meta-analysis studies identified 178 independent variants for depression (*n* = 1,154,267) and 5 for anxiety disorders (*n* = 114,019), with single nucleotide polymorphism (SNP)-based heritabilities of 11% for lifetime depression and 26% for lifetime anxiety disorders [9, 10].

There is a longstanding recognition that in addition to the genetic background, the environment has a major contribution to depression and anxiety disorders. For example, twin studies show that environmental effects account for 0.63 and 0.68 of the phenotypic variance of MDD and generalized anxiety disorder, respectively [7, 8]. Particularly stress-related exposures such as long-term difficulties, stressful life events, childhood trauma, reduced social support, and loneliness, are important risk factors for depression and anxiety disorders [11–13].

However, it is still unclear whether genetic effects are moderated by these stress-related environmental factors. Early studies focused on the interplay between a limited set of biological candidate genes and stress-related exposures [14–17], but this approach has been discontinued as it has yielded few replicable results. As depression and anxiety are polygenic disorders, a better approach is to leverage genome-wide association studies (GWAS) results to capture genetic susceptibility, by using a polygenic risk score (PRS), rather than using a priori chosen candidate genes [18, 19].

However, results from studies using PRSs to study gene-by-stress interactions in relation to MDD have been inconsistent. For example, an Australian study (*n* = 5221) and a study from the UK (*n* = 4919) showed that a PRS by stressful life events interaction effect predicted MDD (both studies only in females) [11, 20], while this was not found in a study from the USA (*n* = 8761) [21]. Similarly, a significant interaction between PRS and childhood trauma on MDD in the UK Biobank has been reported (*n* = 92,957) [12]. However, based on a different statistical approach, a partly different sample and a more broadly defined measure of childhood trauma no significant interaction was found [22]. Finally, in a meta-analysis including 3024 MDD cases and 2741 controls from nine cohorts additive significant effects of PRS and childhood trauma on MDD were found, but no interaction [23]. In addition, no interaction effect was observed between PRS and reduced social support on depressive symptoms (*n* = 5221) [11]. It is likely that these inconsistent findings are due to small sample sizes, or PRSs based on still relatively small GWAS discovery samples. GWAS studies of anxiety have so far been smaller than for MDD, and the lower power of GWASs of anxiety may explain why no studies so far have investigated the interaction between PRS and stress-related exposures on anxiety.

In a large population-based cohort study (*N* = 41,810), we calculated PRSs for depression and anxiety based on recent large GWASs [10, 24]. Our aim was to investigate whether genetic effects on depression and anxiety were moderated by a comprehensive set of stress-related exposures, including long-term difficulties, stressful life events, reduced social support, childhood trauma, and loneliness.

## Methods

### Study sample and design

We used data from the ongoing Lifelines Cohort Study. Lifelines is a prospective population-based cohort study recruiting over 167,000 participants including multi-generation family members in the North of the Netherlands between 2006 and 2013 [25]. Lifelines employs a broad range of investigative procedures in assessing the biomedical, socio-demographic, behavioral, physical and psychological factors which contribute to the health and disease of the general population, with a special focus on multimorbidity and complex genetics [25]. Among all participants, genome-wide genetic data of over 50,000 participants are available [25]. The Lifelines Cohort Study is conducted according to the principles of the Declaration of Helsinki and in accordance with the research code of University Medical Center Groningen, and is approved by its medical ethical committee. All participants signed an informed consent form.

## Measurements

### Outcomes

Current depression and anxiety were measured using the MINI International Neuropsychiatric Interview (MINI) [26] for adults. The MINI was performed as an individual face-to-face interview by a trained research nurse at baseline when participants visited a Lifelines research facility. During the follow-up, the MINI was administered as a digital questionnaire with participants entering their answers under the supervision of a trained research nurse on location. In the early stages of the baseline measurement wave, “skips” were used in the MINI interview such that some questions were asked, or not asked, depending on the participants’ responses on screening questions. In order to collect complete data on all participants, skips were removed from the MINI at a later stage of the baseline measurement. To capture anxiety and depression as a continuous trait using sum scores, we used the MINI without skips at the second assessment for participants who had been assessed using the MINI with skips at baseline. We used 10 items in the MINI to calculate the sum scores for depression and 10 for anxiety. The sum score of anxiety captured four types of anxiety, but mostly GAD in the past six months measured by seven items. In addition, there was one item for panic disorder in the past month, one item for agoraphobia in the past month, and one item for social anxiety disorder in the past month. For children, depression and anxiety were measured by combining the Child Behavior Checklist (CBCL) [27] and the Youth Self-Report questionnaires (YSR) [28] at baseline, where 13 depression-related items and 6 anxiety-related items were used to calculate the sum score of depression and anxiety. (Details provided in Supplementary).

### Stress-related exposures

For adults, long-term difficulties in the past year were assessed at baseline using the Long-term Difficulties Inventory (LDI) [29]. The LDI is a self-report questionnaire, consisting of 12 items referring to different aspects of life, including housing, work, social relationships, free time, finances, health, school/study, and religion [29]. Each item has a three-point scale: 0=not stressful, 1=slightly stressful, 2=very stressful. Item scores are summed to derive total scores for the LDI, ranging from 0 to 24 points. For children, long-term difficulties were measured at baseline by parent-report using 13 items of the influence of long-term difficulties inventory [30]. Each item has a four-point scale: 0=none, 1=a bit, 2=quite a lot, 3=very much, with the total sum score of long-term difficulties for children ranging from 0 to 39 points. Sum scores of long-term difficulties for children were converted to the same scale as for adults (0–24 points).

For adults, stressful life events in the past year were assessed at baseline using the List of Threatening Events (LTE) [29]. The LTE is a 12-item self-report questionnaire, comprising 12 major categories of stressful life events with established long-term consequences [29]. Participants answered whether or not each item occurred (0=no, 1=yes), with the total sum score ranging from 0 to 12 points. For children, stressful life events in the past 2 years were measured at baseline by parent-report questionnaires with 13 items relevant to whether the stressful event occurred (0=no, 1=yes) [30]. The sum scores of stressful life events for children were converted to the same scale as for adults (0–12 points).

For adults, social support was assessed at baseline using the 9 items short version of the Social Production Function Instrument for the Level of well-being (SPF-IL) [31]. Each item has a 4-point scale (range 0–3), with the total sum score ranging from 0 to 27 points. For children, social support was measured by combining the 8-item self-report with the parent-report PROMIS-29 Profile at the second assessment [32]. Each item has a 4-point scale (range 0–4), with a total sum score range of 0–32 points. The sum scores of social support for children were converted to the same scale as for adults (0–27 points).

Childhood trauma was measured only among adults using a 28-item retrospective self-report Childhood Trauma Questionnaire-Short Form (CTQ-SF) [33]. The CTQ-SF was administered approximately 2 years after the second assessment in Lifelines and measures traumas experienced in childhood as a total score and as five dimensions: emotional abuse, emotional neglect, physical abuse, physical neglect, and sexual abuse. There are 5 items on each scale of the CTQ-SF. Each item has a five-point scale: 1=never true, 2=rarely true, 3=sometimes true, 4=often true, and 5=very often true. We calculated the total sum score of childhood trauma (25–125 points), and the sum score of each subscale (5–25 points).

Loneliness was also measured only among adults 2 years after the second assessment using the 6-item De Jong Gierveld Loneliness Scale [34]. Each item is scored from 1 to 5 to reflect how much loneliness is experienced (no!, no, more or less, yes, yes!), with the total sum score ranging from 6 to 30 points.

### Genetic data

Genome-wide genotyping was available for 55,063 participants. The first subset of 17,033 participants was genotyped using the Illumina CytoSNP-12v2 array [25]. Pre-imputation quality control was performed in which samples and variants were excluded with a call rate <95%, as well as variants with Hardy-Weinberg equilibrium (HWE) *P* < 1 × 10^–4^, or minor allele frequency (MAF) < 1%, and samples with a sex mismatch, deviating heterozygosity (>4 SD from the mean) or of non-European ancestry. A total of 15,400 samples and 265,000 SNPs were available for analysis. The second subset of 38,030 participants was genotyped using the Infinium Global Screening Array® (GSA) MultiEthnic Disease Version [25]. Standard quality control was performed on both samples and markers, including removal of samples and variants with a low genotyping call rate (<99%), variants showing deviation from HWE (*P* < 1 × 10^–6^) or excess of Mendelian errors in families (>1% of the parent-offspring pairs), and samples with a sex mismatch, and very high or low heterozygosity. After quality control, a total of 36,339 samples and 571,420 SNPs were available for analysis. These two genotyping datasets were imputed using the HRC panel v1.1 at the Sanger imputation server [35], and variants with an imputation quality score higher than 0.4 for variants with a MAF > 0.01 were retained. After removing duplicate samples between the two genetic datasets (*n* = 937), 50,802 participants with genetic data were available. (Supplementary Fig. S1).

### Polygenic risk scores

PRSs were generated by PLINK v1.9 [36] and R 3.5.2 [37], and were calculated using the GWAS data of the Lifelines participants and summary statistics of recent large GWAS meta-analyses for depression [24] and anxiety [10]. PLINK removed strand-ambiguous SNPs and pruned our target sample to obtain independent SNPs using clumping (*r*^2^ = 0.1, within a 1000 kb window). Independent risk alleles in dosage were weighted by the allelic effect sizes estimated in the summary statistics and aggregated into PRSs in R 3.5.2. PRSs were generated for eleven *P* thresholds: <5 × 10^–8^, <1 × 10^–7^, <1 × 10^–6^, <1 × 10^–5^, <1 × 10^–4^, <0.001, <0.01, <0.05, <0.1, <0.5, and ≤1.0, determined by the summary statistics and standardized. We used the PRSs explaining the largest variance for depression and anxiety as the best-fit PRSs in our main analysis. Further, we performed principal component analysis (PCA) on the total set of 11 PRSs, and used the first PRS-PC in sensitivity analysis [38].

### Statistical analysis

Linear mixed regression models were used to estimate the variance in depression and anxiety scores explained by PRS, stress-related exposures, and their interactions with adjustment for relatedness between individuals. Age, sex, chips (CytoSNP or GSA), and 10 principal components were included as covariates.

For each of the five stress-related exposures, we used the following linear mixed regression models to assess the effects of PRS, stress-related exposures and their interactions, with model 3 capturing our main research question:

**Model 1 (main effects of PRSs):** Depression/Anxiety scores = *β*_*0*_ + *β*_*1*_ *PRS* + *Covariates*

**Model 2 (main effects of stress-related exposures):** Depression/Anxiety scores = *β*_*0*_ + *β*_*1*_ *stress-related exposure* + *Covariates*

**Model 3 (full interaction model):** Depression/Anxiety scores = *β*_*0*_ + *β*_*1*_ *PRS* + *β*_*2*_ *stress-related exposure* + *β*_*3*_ *PRS* *×* *stress-related exposure* + *Covariates*

**Model 4 (full model** + **SES):** Depression/Anxiety scores = *β*_*0*_ + *β*_*1*_ *PRS* + *β*_*2*_ *stress-related exposure* + *β*_*3*_ *PRS* *×* *stress-related exposure* + *β*_*4*_ *SES*_*1–4*_ + *Covariates*

**Model 5 (model 4** + **SES** *×* **stress-related exposure):** Depression/Anxiety scores = *β*_*0*_ + *β*_*1*_ *PRS* + *β*_*2*_ *stress-related exposure* + *β*_*3*_ *PRS* *×* *stress-related exposure* + *β*_*4*_ *SES*_*1–4*_ + *β*_*5*_ *SES*_*1–4*_ *×* *stress-related exposure* + *Covariates*

Recent work by Akimova et al. [39] indicates that the presence of gene-environment correlation (rGE) [40] (i.e., between the depression/anxiety PRS and stress-related exposures [12]) may yield biased results of particularly the main effects in the presence of unobserved confounders. Adjustment for such confounding would resolve this and allow for an estimation of the magnitude of the bias [39]. Thus we calculated Pearson’s correlations between PRSs of anxiety and depression and stress-related exposures, and additionally explored if the findings from our main analysis were robust against adjustment for socio-economic status (SES) as a major potential confounder of the relation between stress exposures and depression/anxiety in model 4 [41]. To comprehensively adjust for SES we added a total of four SES variables (educational attainment, occupational status, disposable household income, and neighborhood SES) to the model. Further simulation analyses by Akimova et al. [39] revealed that interactions between unobserved confounders and environmental exposures may inflate the effect of gene-by-environment interaction (G × E) when not taken into account. Therefore, we also included the interactions between stress-related exposures and all four SES indices in model 5 to test whether and to what extent these stress × SES interactions had inflated the effect of PRS × stress on depression and anxiety. In addition, with the addition of each additional predictor, model fit improvement was checked based on *R*^2^*s* and F-tests (details in the supplementary).

As sum scores of depression and anxiety have skewed distributions, in order to check whether interaction effects were dependent on the distribution of the outcome, we conducted sensitivity analyses for model 3 by normalizing the outcome variables. This was accomplished by fitting a model with covariates (including age, sex, chips, and 10 principal components) to the data and saving the residuals of anxiety and depression, followed by performing an inverse normal transformation on these residuals, which pulls in the right tail and introduces a left tail. The resulting distribution is approximately normal and useful for robustness checks, but note, however, that the original scale is truer to reality. That is, psychopathology in the general population is inherently skewed and the score variation in the right tail is meaningful, representing the degrees of symptom severity that we aim to measure.

Childhood trauma and loneliness were measured 2 years after the second assessment (*n* = 20,152); therefore, we used outcome measures at the second assessment (*n* = 18,635) supplemented by sum scores of depression and anxiety at baseline for participants who did not have measurements of depression and anxiety at second assessment (*n* = 1517). As the measurement instruments of depression, anxiety and stress were different for adults and children, separate analyses for model 3 were conducted in adults and children for LDI, LTE, and social support. In addition, some items used to calculate sum score of depression in the MINI or CBCL or YSR were only related to common symptoms (such as problems with appetite, sleep, fatigue, and concentration) but not related to depressive symptoms. We checked the proportion of participants who had only common symptoms but without any of the core depressive symptoms pertaining to sadness or loss of pleasure, and conducted sensitivity analysis based on participants who had at least one core depression symptom. Attrition analyses were conducted to test differences in demographic characteristics between participants with and without missing data. As a final exploration, we fitted model 3 to the five subscales of childhood trauma.

All parameters from the models were estimated using ASReml-R [42] adjusting for familial relationships in the Lifelines data and the significance of the effects (*β*) was assessed by the Wald test. We corrected for multiple testing using the false discovery rate (FDR < 0.05) corrected for 55 tests (11 PRSs *×* 5 stress-related exposures).

## Results

Among all participants of Lifelines, 41,810 participants provided information on both genetic data and depression and/or anxiety scores (detailed in Supplementary Fig. S1). The characteristics of the participants are in Table 1. Table S1 shows the characteristics of the participants separately for adults and children. Table S2 shows that the gender distribution was comparable between participants with and without missing data, while younger participants had more missing data on social support, childhood trauma, and loneliness.

**Table 1:** Characteristics of participants (*n* = 41,810).

Variables	*n*	Mean±SD/median±IQR/*n* (percent)	Measured waves
Age (years)	41,810	41.95 ± 15.31	Baseline
Gender (female)	41,810	24,640 (58.93%)	
Outcomes			
Sum score of depression	41,524	0.00 (0.00–1.00)	Baseline + second assessment
Sum score of anxiety	41,451	0.00 (0.00–2.00)	
Stress-related exposures			
Long-term difficulties inventory	40,700	2.00 (0.62–3.00)	Baseline
Stressful life events	40,793	1.00 (0.00–2.00)	
Social support	39,875	16.14 ± 3.67	
Childhood trauma	20,100	31.00 (27.00–35.00)	2 years after second assessment
Loneliness	20,105	10.95 ± 3.38	
Socioeconomic status (confounders)			
Educational attainment (years)	41,418	14.34 ± 4.09	Baseline
Disposable household income (euro/month)	37,403	1622.50 ± 510.94	
Occupational status	40,528	44.02 ± 12.94	
Neighborhood SES	40,771	−0.54 ± 1.05	

*SD* standard deviation, *IQR* interquartile range.

Baseline measurements took place during 2007–2013. Second assessment took place during 2014–2017. Childhood trauma and loneliness questionnaires were measured at 2 year after face to face second assessment during 2016–2019.

### Variance explained by stress-related exposures

**Fig. 1:**
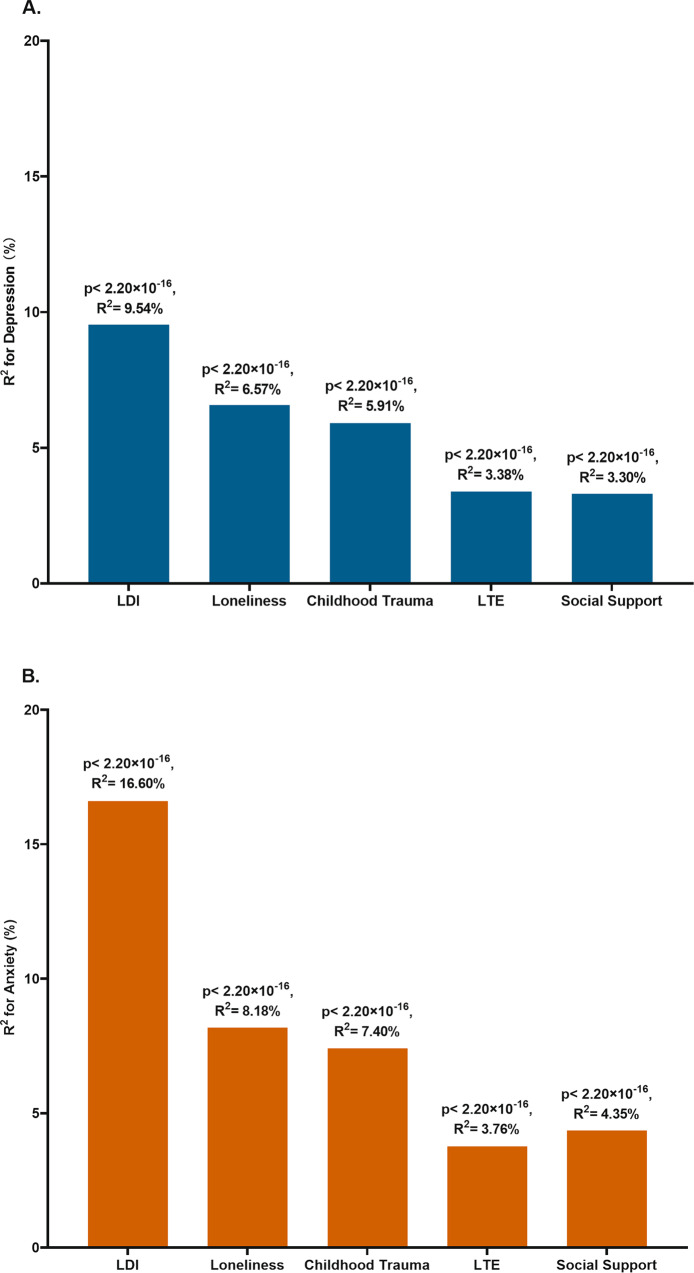
Variance explained by stress-related exposures for depression and anxiety. **A** Variance explained for depression **B** Variance explained for anxiety LDI Long-term difficulties inventory; LTE List of threatening experiences.

Stress-related exposures explained a significant proportion of the variance for depression and anxiety. For depression, the variance explained by stress-related exposures ranged from 3.30% for social support to 9.54% for long-term difficulties (Fig. 1A). For anxiety, the proportion explained by stress-related exposures was higher than that for depression, ranging from 3.76% for stressful life events to 16.60% for long-term difficulties (Fig. 1B).

### PRS and G × E for depression

**Fig. 2:**
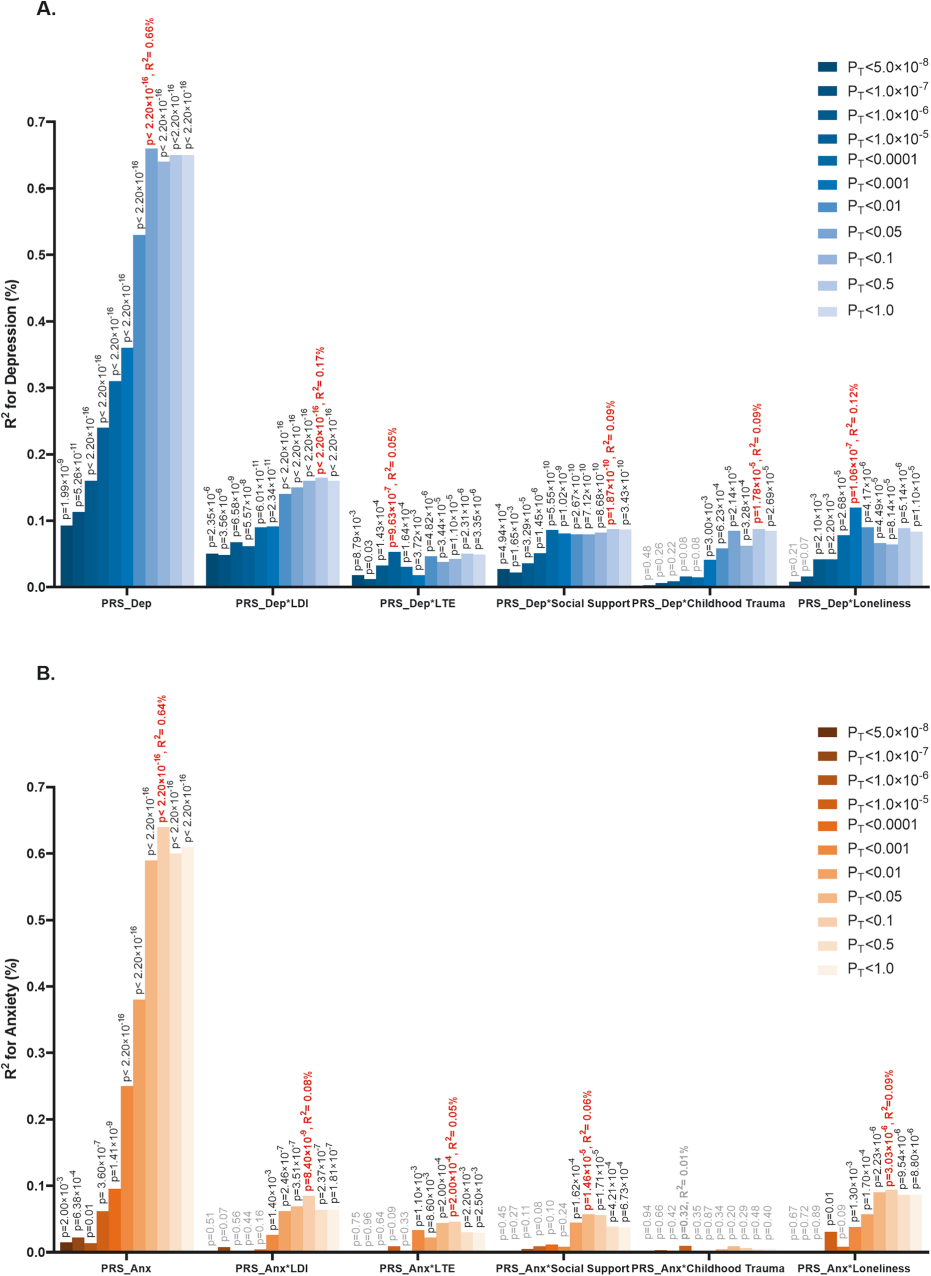
Variance explained by PRSs and PRSs × stress-related exposures for depression and anxiety. **A** Variance explained for depression **B** Variance explained for anxiety. LDI Long-term difficulties inventory; LTE List of threatening experiences. The significance of the 55 interaction tests (11 PRS *×* 5 stress-related exposures) was adjusted for multiple testing using the false discovery rate (FDR < 0.05). For depression, 48 tests were significant (*p*-value in black), and for anxiety, 24 tests were significant (*p*-value in black).

**Fig. 3:**
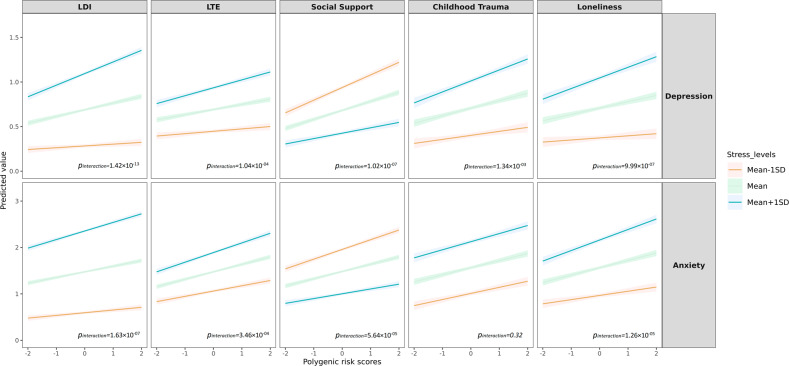
Interaction between PRSs and stress-related exposures for depression and anxiety. DI Long-term difficulties inventory; LTE List of threatening experiences. The PRS used in the interaction plot was at the most significant *p*-thresholds. LDI interacted with PRS_Dep_ at *p*-threshold = 0.5 and PRS_Anx_ at *p*-threshold = 0.1. LTE interacted with PRS_Dep_ at *p*-threshold = 1.0 × 10^–05^ and PRS_Anx_ at *p*-threshold = 0.1. Social support interacted with PRS_Dep_ at *p*-threshold = 0.5 and PRS_Anx_ at *p*-threshold = 0.05. Childhood trauma interacted with PRS_Dep_ at *p*-threshold = 0.5 and PRS_Anx_ at *p*-threshold = 0.05. Loneliness interacted with PRS_Dep_ at *p*-threshold = 0.001 and PRS_Anx_ at *p*-threshold = 0.05.

The PRS for depression had a significant effect on the depression score (*β* = 0.11, *P* < 2.20 × 10^–16^) explaining 0.66% of the variance at its best *P*-threshold (*P*-threshold=0.05; Fig. 2A). We identified significant interactions between the PRS for depression and all five stress-related exposures, with variance explained by G × E ranging from 0.05% for stressful life events to 0.17% for long-term difficulties. The interactions were plotted at their best *P*-threshold (Fig. 3). Higher levels of long-term difficulties, stressful life events, reduced social support, childhood trauma and loneliness amplified the effect of the PRS on depression. For example, the depression score increased 63% as the standardized PRS for depression changed from −2 to 2 for high exposure to long-term difficulties (mean+1SD; blue line of LDI for depression in Fig. 3), while this increase was only 33% for lower exposure to long-term difficulties (mean−1SD; orange line of LDI for depression in Fig. 3). As a second example, lower levels of social support showed a significant interaction with PRS for depression. The depression score increased 88% as the standardized PRS for depression changed from −2 to 2 for lower social support (mean−1SD; orange line of social support for depression in Fig. 3), while this increase was 83% for higher social support (mean+1SD; blue line of social support for depression in Fig. 3).

Sensitivity analysis of the interaction between PRS-PC and stress-related exposures on depression showed similar patterns as the PRS at its best *P*-threshold (Figs. S2, S3). A similar pattern of findings was found for the inverse normally transformed depression scores; although most PRS *×* stress interactions became smaller, they remained significant (Table S3). Table S4 and Fig. S4 show that adjusting for SES in model 4 and additionally for stress × SES interactions in model 5 only led to relatively modest reductions in effect sizes of the PRS *×* stress interactions with all of these interactions remaining significant. Table S5 shows that the total variance explained by stress, PRS, and stress × PRS for depression ranged from 3.56% by LTE and PRS and LTE × PRS, to 9.69% by LDI and PRS and LDI × PRS. With the addition of each predictor, the variance explained for depression (i.e., *R*^2^) increased, and the F-tests for model comparison was significant (Table S5). Separate analyses in children and adults were overall consistent (Table S6). For the depression sum score, we found that 21.08% of participants with a non-zero score had only common symptoms but no core depressive symptoms. Compared with total depression phenotype, after removing these participants with only common symptoms, the effect sizes of PRS, stress, and their interactions increased, and more variance of depression was now explained by these predictors (Table S7). Table S8 shows that correlations between PRSs for depression and stress-related exposures were small but significant (ranging from −0.04 to 0.08). Finally, the interactions between PRSs and 5 subscales of childhood trauma are shown in Fig. S5 and Table S9. Emotional abuse, emotional neglect, physical abuse, and physical neglect significantly amplified the genetic effects on depression, while no interaction was found for sexual abuse.

### PRS and G × E for anxiety

The PRS for anxiety had a significant effect on the anxiety score (*β* = 0.19, *p* < 2.20 × 10^–16^) explaining 0.69% of the variance (Fig. 2B). We detected significant interaction between the PRS for anxiety and long-term difficulties, stressful life events, reduced social support, and loneliness, but not for childhood trauma. The G × E effects for anxiety were less significant than those for depression, with the variance of anxiety explained by G × E ranging from 0.05% for stressful life events to 0.10% for loneliness (Fig. 2B). Higher levels of long-term difficulties, stressful life events, reduced social support and more loneliness amplified the genetic effects on anxiety (Fig. 3). For example, the anxiety score increased 53% as the standardized PRS for anxiety changed from −2 to 2 for high loneliness levels (mean+1SD; blue line of loneliness for anxiety in Fig. 3), while this increase was 46% for lower loneliness exposure (mean−1SD; orange line of loneliness for anxiety in Fig. 3).

Sensitivity analysis of the interaction between PRS-PC and stress-related exposures on anxiety showed a similar pattern compared with the PRS at its best P-threshold (Figs. S2, S3). Inverse normally transformed anxiety scores yielded a similar pattern of findings as was found using the original anxiety scales in model 3, and were still significant (Table S3). Similar as for depression Table S4 and Fig. S4 show that after adjusting for 4 SES variables (model 4) and additionally for stress × SES interactions in model 5 only led to relatively modest reductions in effect sizes of the PRS *×* stress interactions with all significant interactions (i.e., not including childhood trauma) remaining significant. Table S5 shows that the total variance explained by stress, PRS, and stress × PRS for anxiety ranged from 4.29% by LTE and PRS and LTE × PRS, to 16.56% by LDI and PRS and LDI × PRS. With the addition of each predictor, the variance explained for anxiety (i.e., *R*^2^) increased, and the F-tests for model comparison was significant, except for the interaction between childhood trauma and PRS for anxiety (Table S5). Table S8 shows that correlations between PRSs for anxiety and stress-related exposures were small but significant (ranging from −0.03 to 0.07). Figure S5 showed physical abuse and sexual abuse amplified the genetic effects on anxiety, while no interaction was found for the other 3 subscales of childhood trauma.

## Discussion

In this large and comprehensive G × E study for depression and anxiety, we showed that reduced social support and higher levels of long-term difficulties, stressful life events, and loneliness amplified polygenic risk for both depression and anxiety. This was also found for childhood trauma in relation to depression, but not in relation to anxiety. We showed further that stress-related exposures explained more variance in anxiety than depression, that PRSs explained similar variance in anxiety (0.64%) and depression (0.66%) and that interactions between PRSs and stress-related exposures explained more variance in depression than anxiety.

Interactions between PRSs and stress-related exposures for depression and anxiety in the present study were highly consistent, compared with inconsistent findings in previous studies [11, 21, 23, 43]. Inconsistent findings are likely due to the small effect size of the interaction effects, combined with much smaller sample sizes in previous studies compared to our current sample size. In addition, the quality and sample size of GWAS studies is steadily improving, and with that the PRSs have improved as well [44]. Thus, our study provides robust evidence on the presence of polygenic risk-by-environment interactions in relation to depression and anxiety. Epigenetics may offer one possible molecular mechanism underlying interactions between PRSs and stress-related exposures. A systematic review showed that epigenetic changes constitute a key mechanism in the interaction of stress-related exposures with the genome leading to stable changes in DNA structure and gene expression [45]. In particular, DNA methylation at multiple CpG sites in stress-related genes (e.g., *NRC31*, *SLC6A4*, and *BDNF*) was associated with depression and partially mediated the association between childhood maltreatment and depression [45]. While our findings offer evidence on polygenic risk-by-environment interactions, the value of our findings for clinical screening for individuals with both high genetic susceptibility and exposure to high stress level is very limited, given the small effects. Potentially, with still improved GWASs of depression and anxiety in the future, interaction effects of PRS and stress exposures (in addition to their main effects) may become useful as part of multivariable prediction algorithms [44].

Stress-related exposures explained more variance in anxiety than depression, with a potential explanation that our measure of depression largely represented the past two weeks while that of anxiety the past 6 months. In addition, a previous study conducted among the family members of patients with heart failure also found a higher correlation between stressful life events and anxiety (0.66) than between stressful life events and depression (0.53) [46]. On the other hand, interactions between PRSs and stress-related exposures explained more variance in depression than in anxiety which suggests that an explanation based on the two weeks versus 6 months timeframe for depression and anxiety, respectively, is too simple. Given the absence of previous studies on PRS and stress-related exposure interactions on anxiety, it is difficult to embed the finding in previous literature. Also, it is unknown whether this stronger interaction effect for depression is mirrored at the epigenetic level. We found some indications of different G × E effects for the subtype of childhood trauma in relation to anxiety and depression, i.e., emotional abuse, emotional neglect, physical abuse and physical neglect for depression, and physical abuse and sexual abuse for anxiety. In all, future studies need to determine if the current differences in findings replicate, and more generally we conclude that more precise knowledge of PRS and stress-related exposures in relation to anxiety and depression is needed. Furthermore, as depression and anxiety were found to be highly genetically correlated in our own data (0.94 [47]) as well as elsewhere (0.79 [48]), future studies need to focus on the question of whether stress-related exposures moderate genetic susceptibility to these two conditions at a higher aggregated genetic level (i.e., shared genetics, for example, modeled by means of genomic structural equation modeling [49]) or more at the genetic levels unique to depression and anxiety.

Gene-by-environment interactions focus on the joint effects of genetic and environmental factors on the variation of the phenotype. However, these effects are often not independent. An individual’s genetic make-up may influence the environment they are exposed to, i.e., gene-environment correlation (rGE) [40], which might confound gene-environment interactions (G × E) [50]. A recent simulation study showed that higher values of rGE lead to underestimation of the genetic (i.e., PRS) main effect [39]. Importantly, G × E showed no inflation in the presence of high rGE [39]. In addition, small rGEs were found in the present study (Table S8). Likewise, in the present study, following simultaneous adjustment for four measures of socio-economic status as potential confounders, the interaction effects between stress-related exposures and PRSs only attenuated slightly and remained significant (Table S4 and Fig. S4). Furthermore, interactions between potential confounders and environmental exposures may inflate the effect of G × E when not properly accounted for [39]. In line with this, after adjusting for the interactions between SES and stress-related exposures, G × E effects decreased. However, all G × E effects remained significant (Table S4), confirming the consistency of the interactions between PRSs and stress-related exposures for depression and anxiety.

The following limitations of our study need to be considered. First, while our measures were adequate, they also had some limitations. The depression score was largely based on symptoms during the past two weeks, which is a rather short period. The anxiety score was mostly based on GAD symptoms: seven items were scored on the basis of the past six months. Added to these were single item questions for panic disorder, agoraphobia, and social anxiety disorder. Thus, results should be interpreted mostly with GAD in mind. Further, although anxiety disorders generally tend to be rather stable (e.g., stability (%) ranged from 53.7% for panic disorder with agoraphobia to 78.9% for social anxiety disorder after 6 years follow-up in a recent study) [51], there was some inconsistency such that single items were scored for a shorter period, i.e., the past month. Therefore, it is likely that we have underestimated G × E effects compared to assessment of the lifetime presence of depression and anxiety disorders. Second, although depression and anxiety and stress exposures were not always measured at exactly the same timepoint, this held for the more stable stress exposures (i.e., retrospectively reported childhood trauma and loneliness) and we tried to accommodate for this as much as possible. For example, for the analyses of the later-in-time collected information on childhood trauma and loneliness, we also used depression and anxiety measures at the second assessment for the majority of participants. To the extent that timing differences had an influence, current effects are underestimated. Third, depression, anxiety, and stress-related exposures had different measurement instruments in adults and children. Combining different measurements for adults and children increased the sample size, but at the same time introduced (some) heterogeneity for phenotypes, which might have reduced effect sizes. Fourth, the distributions of sum scores of depression and anxiety were skewed, which is inconsistent with the assumption of linear mixed modeling. Furthermore, G × E interactions, such as the effect of trauma exposure on depression, are sometimes found to be scale dependent [12]. However, when we applied the inverse normal transformation to the covariate-adjusted residualised scores of depression and anxiety to check the robustness of the findings in sensitivity analyses, we showed that the interactions were still significant (Table S3). While our findings were based on a very large sample, it may be the case that for the rarest exposure of physical abuse (studied in secondary analyses), we may not have been able to identify a robust G × E interaction effect, as we found a counterintuitive estimate for anxiety (*β* = −0.04, *P* = 0.02). This finding emphasizes that the identification of robust G × E effects requires large sample sizes, especially if exposures are rare. The past has seen a large extent of non-replicated G × E interaction effects, and while this may have been primarily due to the now abandoned candidate gene approach [14–17]. Insufficient sample size may have played a role in those studies as well, and thus, to avoid false positive results [52], a continued warning on the need for large sample sizes for robust G × E effects in current, PRS based, G × E studies remains of strong importance. Despite the aforementioned limitations, most of which revolve around finding smaller effects due to design features, our study nonetheless showed highly consistent G × E effects.

In summary, the present study provides consistent evidence on the enhancement of genetic risk by stress-related exposures on depression and anxiety. We expect that future studies focusing on lifetime depression and anxiety, and using PRSs based on larger (future) GWAS discovery samples may reveal even stronger interaction effect sizes. While currently not useful in clinical practice, it is plausible that with expected improvements of depression and anxiety GWASs in the future, interactions effects of PRS and stress-related exposures may become useful as part of multivariable prediction algorithms.

## Supplementary information

Supplementary materials
