# Supplementary material for: Stress-related exposures amplify the effects of genetic susceptibility on depression and anxiety
Source: Transl Psychiatry. 2023 Jan 30;13:27. doi: 10.1038/s41398-023-02327-3 (PMC9886926; doi:10.1038/s41398-023-02327-3)
Supplement: Supplementary file 1 — Supplementary materials [file 41398_2023_2327_MOESM1_ESM.docx]

**Stress-related exposures amplify the effects of genetic susceptibility on depression and anxiety**

**Supplementary Materials**

**Part 1: Supplementary Results**

**Figure.S1 Flow chart** CytoSNP, Illumina CytoSNP-12v2 array; GSA, Infinium Global Screening Array® (GSA) MultiEthnic Disease Version.

***
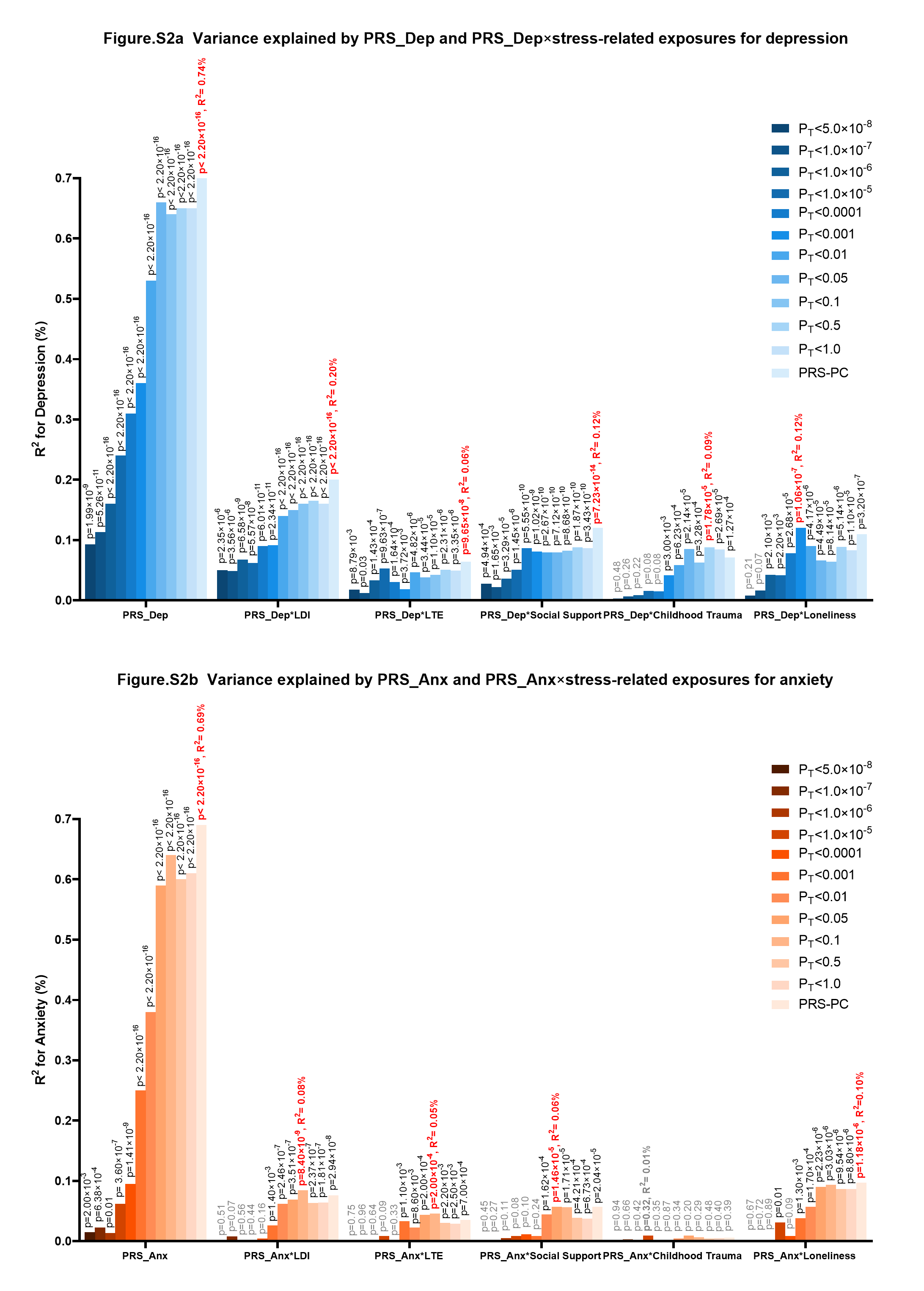
***

**Figure.S2 Variance explained by PRS-PC and PRS-PC × stress-related exposures for depression and anxiety** LDI, Long-term of difficulties inventory; LTE, List of threatening experiences. Significance of the 60 interaction tests (12 PRS × 5 stress-related exposures) were adjusted for multiple testing using the false discovery rate (FDR<0.05). For depression, 53 tests were significant (*p*-value in black), for anxiety, 28 tests were significant (*p*-value in black).

***
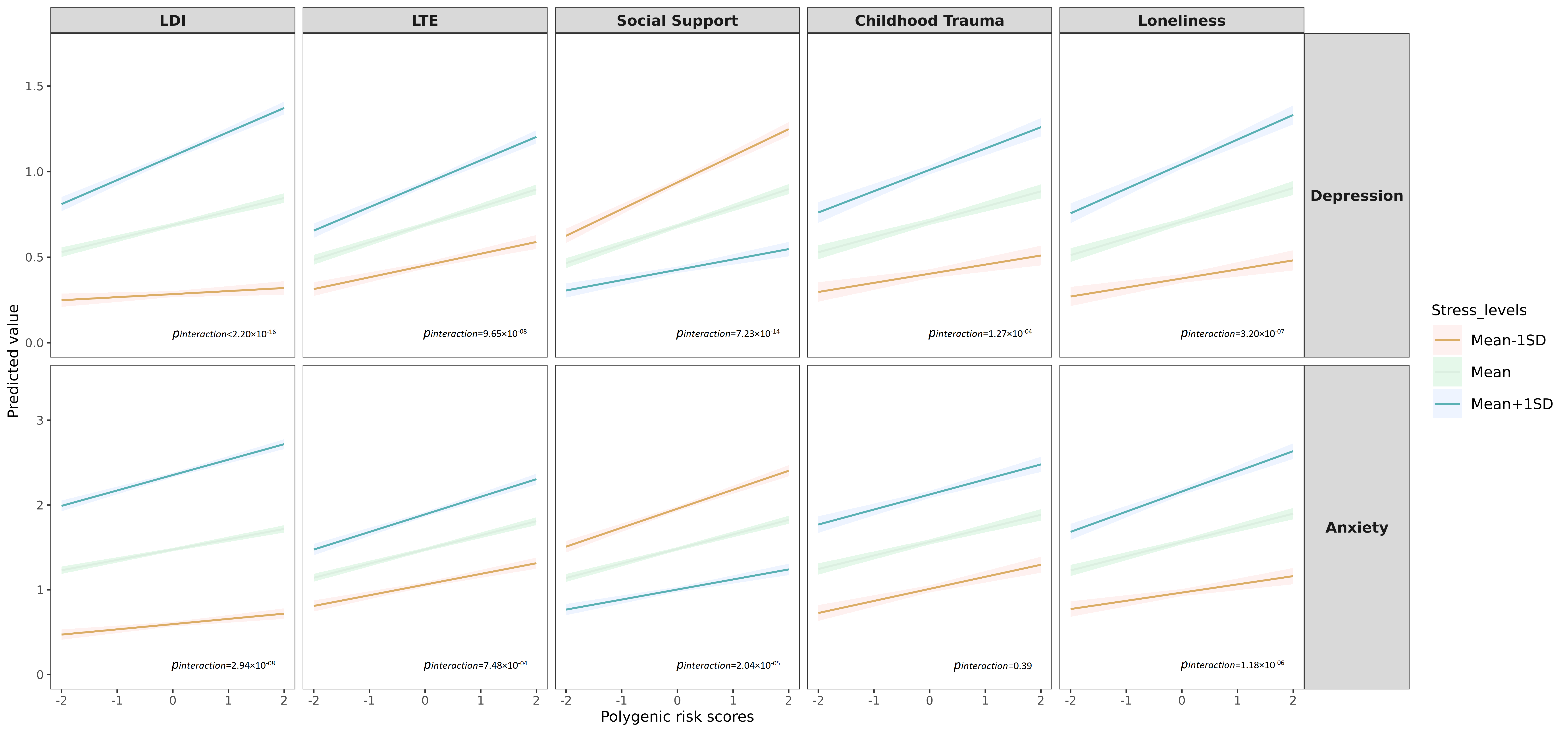
***

**Figure.S3 Interaction between PRSs-PC and stress-related exposures for depression and anxiety** LDI, Long-term of difficulties inventory; LTE, List of threatening experiences.


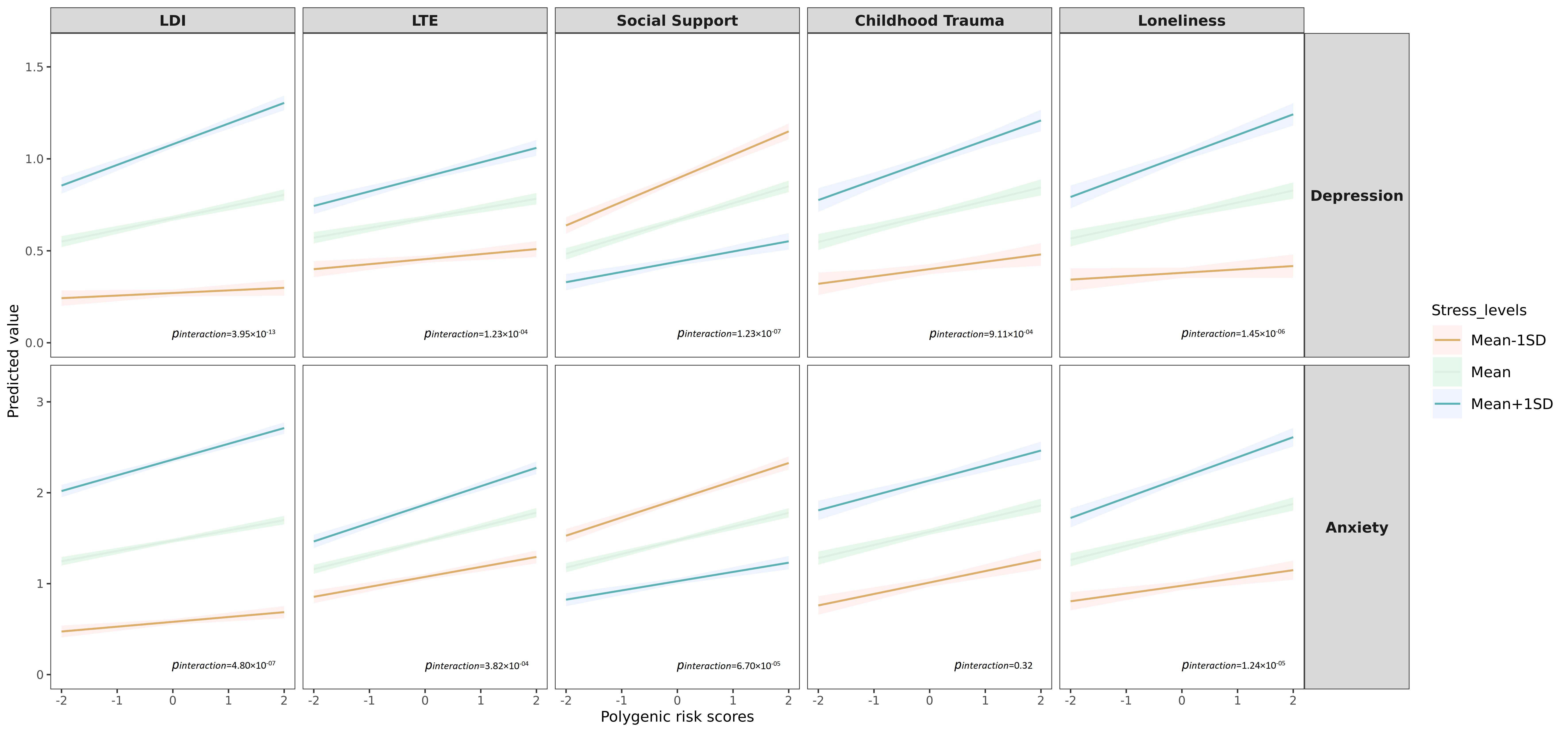


**Figure.S4 Interaction plot of polygenic risk score and stress-related exposures for depression and anxiety after adjusting for socio-economic status**  LDI, Long-term of difficulties inventory; LTE, List of threatening experiences. The PRS used in the interaction plot was at the most significant p-thresholds. LDI interacted with PRS_Dep_ at p-threshold=0.5 and PRS_Anx_ at p-threshold=0.1. LTE interacted with PRS_Dep_ at p-threshold=1.0×10^-05^ and PRS_Anx_ at p-threshold=0.1. Social support interacted with PRS_Dep_ at p-threshold=0.5 and PRS_Anx_ at p-threshold=0.05. Childhood trauma interacted with PRS_Dep_ at p-threshold=0.5 and PRS_Anx_ at p-threshold=0.05. Loneliness interacted with PRS_Dep_ at p-threshold=0.001 and PRS_Anx_ at p-threshold=0.05. After adjusting for 4 social economic status (including educational years, disposable income, occupational status and neighborhood SES), interaction between PRS and stress-related exposures were still significant.


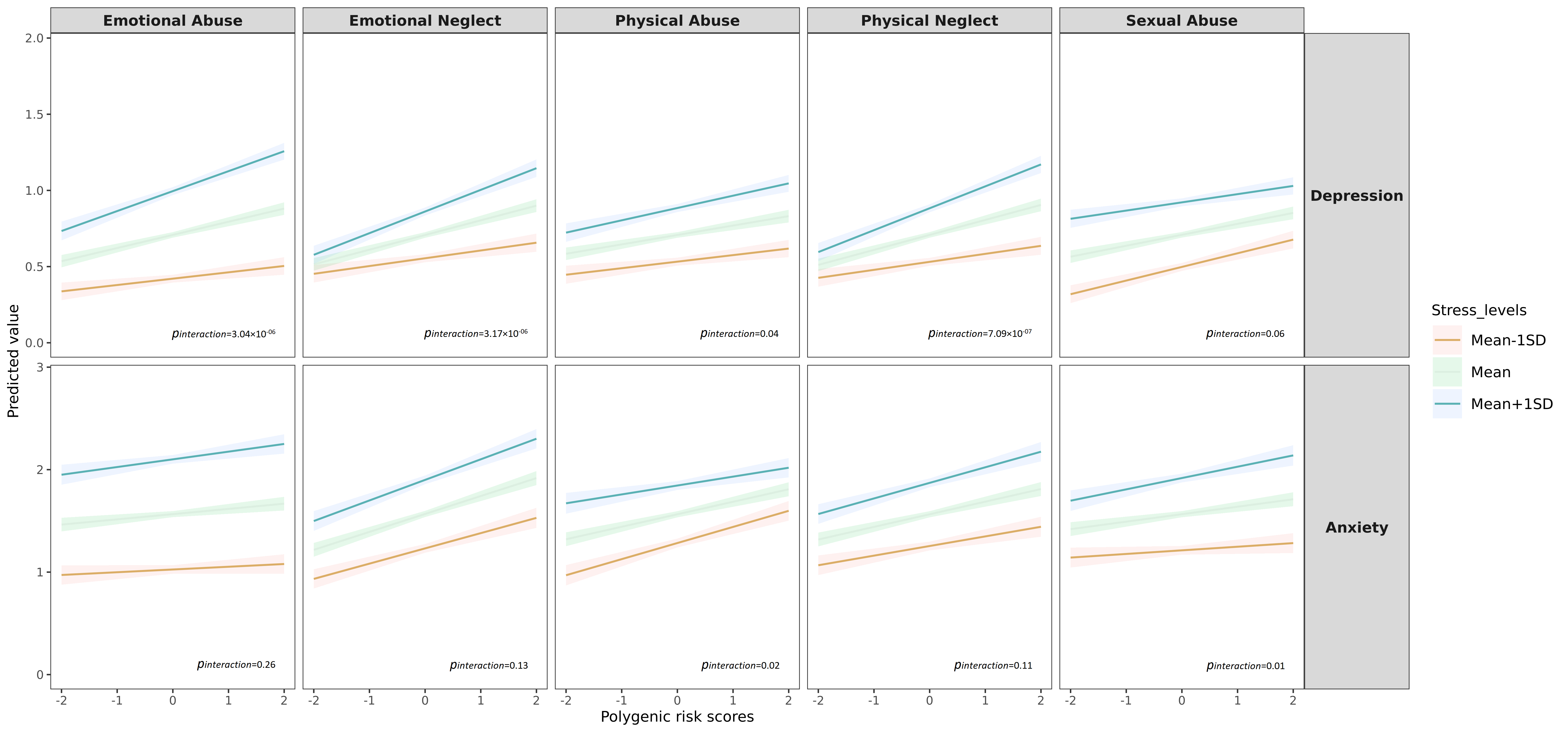


**Figure.S5 Interaction plot of polygenic risk score and 5 subscales of childhood trauma for depression and anxiety** The PRS used in the interaction plot was at the most significant p-thresholds. Emotional abuse interacted with PRS_Dep_ at p-threshold=0.05 and PRS_Anx_ at p-threshold=0.0001. Emotional neglect interacted with PRS_Dep_ at p-threshold=0.01 and PRS_Anx_ at p-threshold=0.05. Physical abuse interacted with PRS_Dep_ at p-threshold=1.0×10^-05^ and PRS_Anx_ at p-threshold=0.001. Physical neglect interacted with PRS_Dep_ at p-threshold=1.0 and PRS_Anx_ at p-threshold=0.001. Sexual abuse interacted with PRS_Dep_ at p-threshold=0.0001 and PRS_Anx_ at p-threshold=1.0×10^-05^.


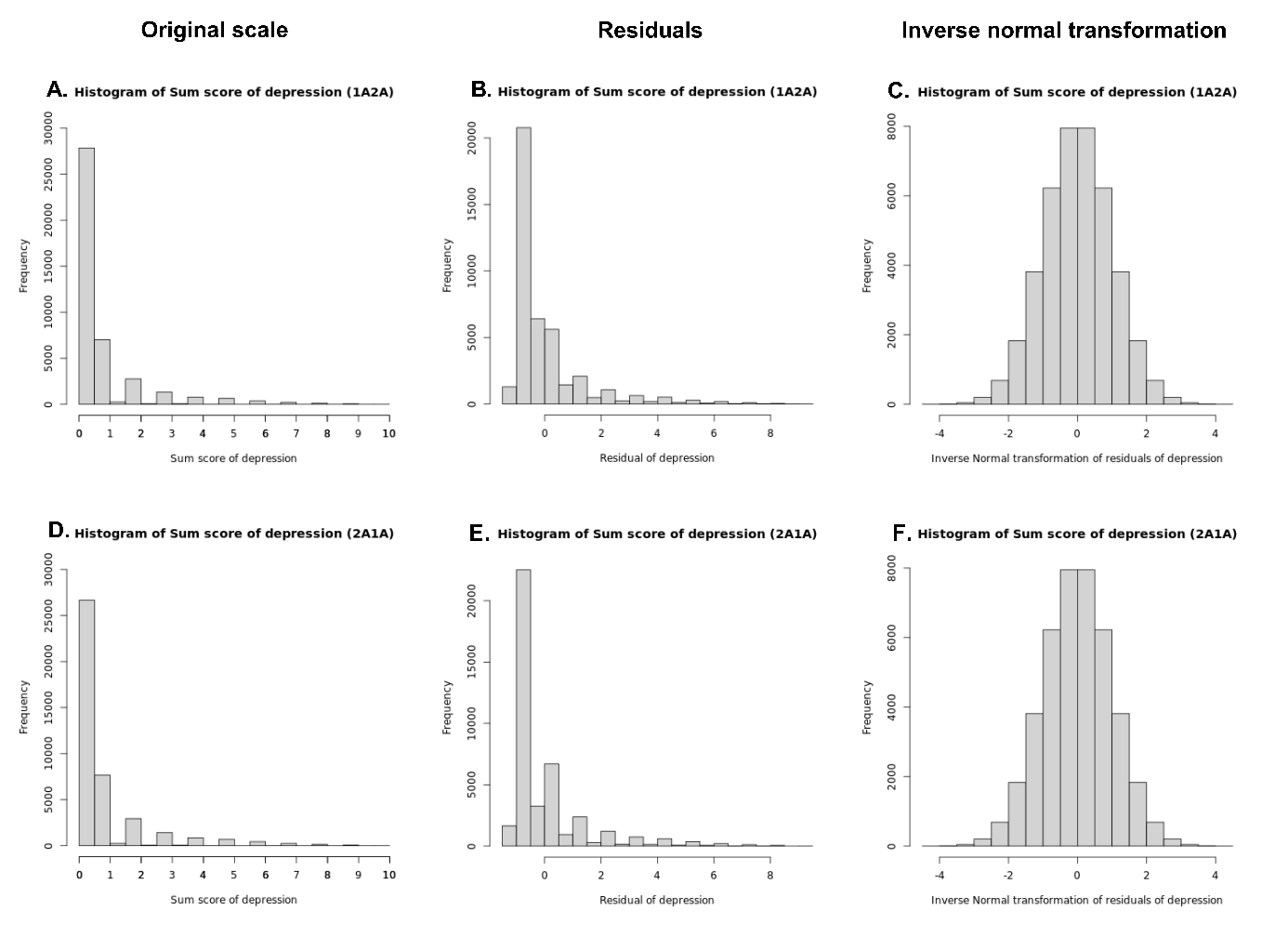


**Figure.S6 Distribution of sum score of depression in different scales** Residuals were generated from the regression model adjusting for age, sex, chip (CytoSNP or GSA) and 10 principal components. Depression (1A2A) represents sum scores mainly measured by MINI at baseline (1A), and for those who didn’t have sum score of depression at baseline (because of skips or not available, n=19,992), we used the sum score of depression at follow-up (2A). As childhood trauma and loneliness were only measured at follow-up (n=20,152), we preferentially used sum scores of depression measured at follow-up as outcome (n=18,635) supplemented by sum scores of current depression at baseline for participants who did not have measurements of depression at follow-up (n=1,517).

***
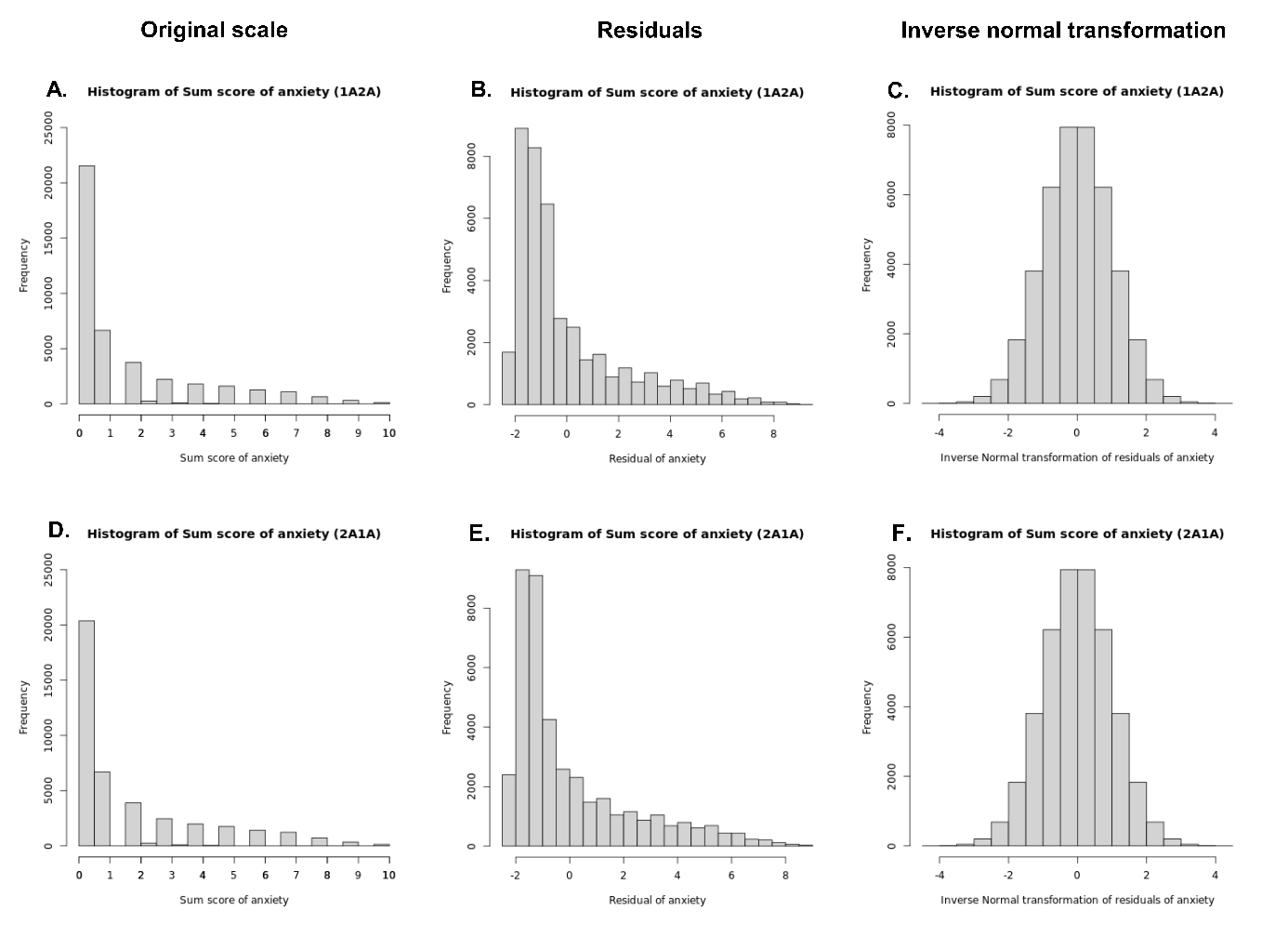
***

**Figure.S7 Distribution of sum score of anxiety in different scales** Residuals were generated from the regression model adjusting for age, sex, chip (CytoSNP or GSA) and 10 principal components. Anxiety (1A2A) represents sum scores mainly measured by MINI at baseline (1A), and for those who didn’t have sum score of anxiety at baseline (because of skips or not available, n=19,992) , we used the sum score of anxiety at follow-up (2A). As childhood trauma and loneliness were only measured at follow-up (n=20,152), we preferentially used sum scores of anxiety measured at follow-up as outcome (n=18,635) supplemented by sum scores of current anxiety at baseline for participants who did not have measurements of anxiety at follow-up (n=1,517).

**Table S1. Characteristics for total sample, adults and children**

| Variables | Wave | Total sample | | Adults | | Children | |
| --- | --- | --- | --- | --- | --- | --- | --- |
|  |  | n | *n/mean ± SD /median(IQR)* | n | *n/mean ± SD /median(IQR)* | n | *n/mean ± SD /median(IQR)* |
| **Age (years)** | Baseline | 41,810 | 41.95±15.31 | 38,945 | 44.12±13.49 | 2,865 | 12.38±2.67 |
| **Gender (female)** |  | 41,810 | 24,640 (58.93%) | 38,945 | 23,199 (59.57%) | 2,865 | 1,441 (50.30%) |
| **Outcomes** |  |  |  |  |  |  |  |
| Sum score of depression | Baseline+ second assessment | 41,524 | 0.00  (0.00-1.00) | 38,660 | 0.00 (0.00-1.00) | 2,864 | 1.00 (0.00-3.00) |
| Sum score of anxiety |  | 41,451 | 0.00  (0.00-2.00) | 38,586 | 0.00  (0.00-2.00) | 2,865 | 1.00 (0.00-2.00) |
| **Stress-related exposures** |  |  |  |  |  |  |  |
| Long-term difficulties | Baseline | 40,700 | 2.00  (0.62-3.00) | 37,962 | 2.00 (1.00-4.00) | 2,738 | 0.00 (0.00-1.00) |
| Stressful life events |  | 40,793 | 1.00  (0.00-2.00) | 38,055 | 1.00 (0.00-2.00) | 2,738 | 0.00 (0.00-1.00) |
| Social support |  | 39,875 | 16.14±3.67 | 38,249 | 15.92±3.47 | 1,626 | 25.42±4.98 |
| Childhood trauma | 2 years after second assessment | 20,100 | 31.00  (27.00-35.00) | 20,100 | 31.00  (27.00-35.00) | -- | -- |
| Loneliness |  | 20,105 | 10.95±3.38 | 20,105 | 10.95±3.38 | -- | -- |
| **Socioeconomic status (confounders)** |  |  |  |  |  |  |  |
| Educational attainment (years) | Baseline | 41,418 | 14.34±4.09 | 38,553 | 14.30±4.13 | 2,865 | 14.91±3.36 |
| Disposable household income (euro/month) |  | 37,403 | 1622.50±510.94 | 34,772 | 1632.28±519.60 | 2,631 | 1493.40±353.83 |
| Occupational status |  | 40,528 | 44.02±12.94 | 37,670 | 43.95±13.11 | 2,858 | 44.93±10.38 |
| Neighbourhood SES |  | 40,771 | -0.54±1.05 | 37,955 | -0.55±1.06 | 2,756 | -0.54±0.96 |

SD standard deviation, IQR interquartile range. Baseline measurements took place during 2007-2013. Second assessment took place during 2014-2017. Childhood trauma and loneliness questionnaires were measured 2 years after the face to face second assessment during 2016-2019. For children, the parameters in above table are in the original scales. The sum score of depression for children ranged from 0 to 18. The sum score of anxiety for children ranged from 0 to 11. Long-term difficulties ranged from 0 to 14. Stressful life events ranged from 0 to 9. Social support ranged from 0 to 32.

**Table S2. Demographic characteristic of participants with and without missing data at baseline**

| Variables | Analyzed data | | | Missing data | | |
| --- | --- | --- | --- | --- | --- | --- |
|  | n (%) | Age  (Mean±SD) | Gender (female) n (%) | n (%) | Age (Mean±SD) | Gender (female) n (%) |
| Outcomes |  |  |  |  |  |  |
| Sum score of depression | 41,524 (99.32) | 41.94±15.33 | 24,455  (58.89) | 286 (0.68) | 42.18±11.75 | 185  (64.69) |
| Sum score of anxiety | 41,451 (99.14) | 41.90±15.31 | 24,413  (58.90) | 359 (0.86) | 47.27±14.08 | 227  (63.23) |
| Stress-related exposures |  |  |  |  |  |  |
| LDI | 40,700 (97.35) | 41.90±15.19 | 23,965  (58.88) | 1,110 (2.65) | 43.75±18.97 | 675  (60.81) |
| LTE | 40,793 (97.57) | 41.90±15.21 | 24,021  (58.89) | 1,017 (2.43) | 43.63±18.72 | 619  (60.87) |
| Social support | 39,875 (95.37) | 43.02±14.49 | 23,596  (59.17) | 1,935 (4.63) | 19.77±14.76 | 1,044  (53.95) |
| Childhood trauma | 20,100 (48.07) | 46.94±13.38 | 12,132  (60.36) | 21,710 (51.93) | 37.32±15.52 | 12,508  (57.61) |
| Loneliness | 20,105 (48.09) | 46.95±13.38 | 12,121  (60.29) | 21,705 (51.91) | 37.31±15.51 | 12,519  (57.68) |

Abbreviations: LDI, Long-term difficulties inventory; LTE, List of threatening experiences.

**Table S3. Interactions between PRSs and stress-related exposures for different scales of depression and anxiety**

| Phenotype | Models | N | Original scales | | Residuals of outcome | | Inverse normal transformation of residuals | |
| --- | --- | --- | --- | --- | --- | --- | --- | --- |
|  |  |  | Beta | *p* | Beta | *p* | Beta | *p* |
| Depression | PRS_dep_0.5*LDI | 40,425 | 0.0541 | <2.00×10^-16^ | 0.0538 | <2.00×10^-16^ | 0.0232 | 5.51×10^-06^ |
|  | PRS_dep_1.0e-5*LTE | 40,521 | 0.0316 | 9.63×10^-07^ | 0.0316 | 9.71×10^-07^ | 0.0127 | 0.0101 |
|  | PRS_dep_0.5*social support | 39,593 | -0.0417 | 1.87×10^-10^ | -0.0420 | 1.35×10^-10^ | -0.0181 | 3.19×10^-04^ |
|  | PRS_dep_0.5*childhood trauma | 20,030 | 0.0384 | 1.78×10^-05^ | 0.0385 | 1.73×10^-05^ | 0.0249 | 2.91×10^-04^ |
|  | PRS_dep_0.001*loneliness | 20,035 | 0.0486 | 1.06×10^-07^ | 0.0496 | 6.24×10^-08^ | 0.0240 | 6.81×10^-04^ |
| Anxiety | PRS_anx_0.1*LDI | 40,354 | 0.0546 | 8.40×10^-09^ | 0.0545 | 9.51×10^-09^ | 0.0182 | 1.89×10^-04^ |
|  | PRS_anx_0.1*LTE | 40,442 | 0.0385 | 2.10×10^-04^ | 0.0385 | 2.12×10^-04^ | 0.0120 | 0.0162 |
|  | PRS_anx_0.05*social support | 39,520 | -0.0452 | 1.46×10^-05^ | -0.0461 | 1.02×10^-05^ | -0.0172 | 5.82×10^-04^ |
|  | PRS_anx_0.05*childhood trauma | 20,010 | 0.0192 | 0.2009 | 0.0218 | 0.1478 | 0.0059 | 0.4051 |
|  | PRS_anx_0.05*loneliness | 20,014 | 0.0708 | 2.23×10^-06^ | 0.0716 | 2.03×10^-06^ | 0.0252 | 4.45×10^-04^ |

Residuals were generated from the regression model adjusting for age, sex, chip (CytoSNP or GSA) and 10 principal components.

**Table S4. Differences in effects of interaction between PRSs and stress-related exposures after adjusting for four SES measures (educational attainment, occupational status, disposable household income, neighborhood SES) and additionally for SES×stress interaction terms for all 4 SES measures.**

| Phenotype | Model | PRS bestfit P-threshold | n | Beta | *p* | R^2^ (%) | Difference in beta (%) | Difference in R^2^  (△, %) |
| --- | --- | --- | --- | --- | --- | --- | --- | --- |
| Depression | PRS_dep*LDI | 0.5 | 34,204 | 0.0491 | 1.42×10^-13^ | 0.143 |  |  |
|  | PRS_dep*LDI_adjSES | 0.5 |  | 0.0477 | 3.95×10^-13^ | 0.135 | 2.85 | 0.008 |
|  | PRS_dep*LDI_adjSES*LDI | 0.5 |  | 0.0434 | 3.26×10^-13^ | 0.111 | 11.61 | 0.032 |
|  | PRS_dep*LTE | 1.0×10^-5^ | 34,281 | 0.0273 | 1.04×10^-04^ | 0.038 |  |  |
|  | PRS_dep*LTE_adjSES | 1.0×10^-5^ |  | 0.0268 | 1.23×10^-04^ | 0.037 | 1.83 | 0.001 |
|  | PRS_dep*LTE_adjSES*LTE | 1.0×10^-5^ |  | 0.0127 | 0.0114 | 0.007 | 53.48 | 0.031 |
|  | PRS_dep*social support | 0.5 | 33,423 | -0.0378 | 1.02×10^-07^ | 0.073 |  |  |
|  | PRS_dep*social support_adjSES | 0.5 |  | -0.0373 | 1.23×10^-07^ | 0.072 | 1.32 | 0.001 |
|  | PRS_dep*social support _adjSES*social support | 0.5 |  | -0.0326 | 1.17×10^-07^ | 0.045 | 13.76 | 0.019 |
|  | PRS_dep*childhood trauma | 0.5 | 16,795 | 0.0314 | 1.34×10^-03^ | 0.063 |  |  |
|  | PRS_dep*childhood trauma_adjSES | 0.5 |  | 0.0323 | 9.11×10^-04^ | 0.067 | -2.87 | -0.004 |
|  | PRS_dep*childhood trauma _adjSES*childhood trauma | 0.5 |  | 0.0312 | 9.00×10^-04^ | 0.062 | 0.64 | 0.001 |
|  | PRS_dep*loneliness | 0.001 | 16,801 | 0.0484 | 9.99×10^-07^ | 0.125 |  |  |
|  | PRS_dep*loneliness_adjSES | 0.001 |  | 0.0474 | 1.45×10^-06^ | 0.121 | 2.07 | 0.004 |
|  | PRS_dep*loneliness_adjSES*loneliness | 0.001 |  | 0.0340 | 2.18×10^-04^ | 0.065 | 29.75 | 0.060 |
| Anxiety | PRS_anx*LDI | 0.1 | 34,167 | 0.0532 | 1.63×10^-07^ | 0.084 |  |  |
|  | PRS_anx*LDI_adjSES | 0.1 |  | 0.0508 | 4.80×10^-07^ | 0.077 | 4.51 | 0.007 |
|  | PRS_anx*LDI_adjSES*LDI | 0.1 |  | 0.0466 | 4.66×10^-07^ | 0.065 | 12.41 | 0.019 |
|  | PRS_anx*LTE | 0.1 | 34,238 | 0.0404 | 3.46×10^-04^ | 0.046 |  |  |
|  | PRS_anx*LTE_adjSES | 0.1 |  | 0.0399 | 3.82×10^-04^ | 0.045 | 1.24 | 0.001 |
|  | PRS_anx*LTE_adjSES*LTE | 0.1 |  | 0.0380 | 3.77×10^-04^ | 0.040 | 5.94 | 0.006 |
|  | PRS_anx*social support | 0.05 | 33,387 | -0.0459 | 5.64×10^-05^ | 0.052 |  |  |
|  | PRS_anx*social support_adjSES | 0.05 |  | -0.0453 | 6.70×10^-05^ | 0.050 | 1.31 | 0.002 |
|  | PRS_anx*social support _adjSES*social support | 0.05 |  | -0.0424 | 6.61×10^-05^ | 0.043 | 7.63 | 0.009 |
|  | PRS_anx*childhood trauma | 0.05 | 16,787 | 0.0163 | 0.3187 | 0.007 |  |  |
|  | PRS_anx*childhood trauma_adjSES | 0.05 |  | 0.0162 | 0.3210 | 0.007 | 0.61 | 0.0002 |
|  | PRS_anx*childhood trauma _adjSES*childhood trauma | 0.05 |  | 0.0129 | 0.3207 | 0.005 | 20.86 | 0.002 |
|  | PRS_anx*loneliness | 0.05 | 16,793 | 0.0713 | 1.26×10^-05^ | 0.090 |  |  |
|  | PRS_anx*loneliness_adjSES | 0.05 |  | 0.0712 | 1.24×10^-05^ | 0.090 | 0.14 | 0.0001 |
|  | PRS_anx*loneliness_adjSES*loneliness | 0.05 |  | 0.0685 | 1.22×10^-05^ | 0.081 | 3.93 | 0.009 |

Abbreviations: LDI, Long-term difficulties inventory; LTE, List of threatening experiences. All models adjusted for age, sex, chip (CytoSNP or GSA) and 10 principal components.

**Table S5. Model fit for PRS, stress and their interaction for depression and anxiety**

| Phenotype | Stress | Models | Best-fit PT | R^2^ (%) | ∆R^2^ (%) | Model comparison | | | |
| --- | --- | --- | --- | --- | --- | --- | --- | --- | --- |
|  |  |  |  |  |  | Model | F-test | df | *p* |
| Depression | LDI | Model 0: Covariates |  | 1.62 |  |  |  |  |  |
|  |  | Model 1: Covariates+LDI |  | 10.83 | 9.22 | Model 1 vs 0 | 4178.60 | 1 | < 2.20×10^-16^ |
|  |  | Model 2: Covariates+LDI+PRS | 0.5 | 11.13 | 9.52 | Model 2 vs 1 | 137.55 | 1 | < 2.20×10^-16^ |
|  |  | Model 3: Covariates+LDI+PRS+LDI×PRS | 0.5 | 11.29 | 9.69 | Model 3 vs 2 | 75.19 | 1 | < 2.20×10^-16^ |
|  | LTE | Model 0: Covariates |  | 1.60 |  |  |  |  |  |
|  |  | Model 1: Covariates+LTE |  | 4.93 | 3.32 | Model 1 vs 0 | 1415.70 | 1 | < 2.20×10^-16^ |
|  |  | Model 2: Covariates+LTE+PRS | 1×10^-05^ | 5.11 | 3.50 | Model 2 vs 1 | 78.70 | 1 | < 2.20×10^-16^ |
|  |  | Model 3: Covariates+LTE+PRS+LTE×PRS | 1×10^-05^ | 5.16 | 3.56 | Model 3 vs 2 | 22.66 | 1 | 1.94×10^-06^ |
|  | SS | Model 0: Covariates |  | 1.61 |  |  |  |  |  |
|  |  | Model 1: Covariates+SS |  | 5.02 | 3.41 | Model 1 vs 0 | 1421.10 | 1 | < 2.20×10^-16^ |
|  |  | Model 2: Covariates+SS+PRS | 0.5 | 5.58 | 3.97 | Model 2 vs 1 | 233.79 | 1 | < 2.20×10^-16^ |
|  |  | Model 3: Covariates+SS+PRS+SS×PRS | 0.5 | 5.67 | 4.06 | Model 3 vs 2 | 36.80 | 1 | 1.32×10^-09^ |
|  | CT | Model 0: Covariates |  | 1.44 |  |  |  |  |  |
|  |  | Model 1: Covariates+CT |  | 6.87 | 5.43 | Model 1 vs 0 | 1166.50 | 1 | < 2.20×10^-16^ |
|  |  | Model 2: Covariates+CT+PRS | 0.5 | 7.25 | 5.81 | Model 2 vs 1 | 81.83 | 1 | < 2.20×10^-16^ |
|  |  | Model 3: Covariates+CT+PRS+CT×PRS | 0.5 | 7.34 | 5.89 | Model 3 vs 2 | 18.90 | 1 | 1.38×10^-05^ |
|  | LS | Model 0: Covariates |  | 1.45 |  |  |  |  |  |
|  |  | Model 1: Covariates+LS |  | 7.60 | 6.16 | Model 1 vs 0 | 1334.30 | 1 | < 2.20×10^-16^ |
|  |  | Model 2: Covariates+LS+PRS | 0.001 | 7.88 | 6.44 | Model 2 vs 1 | 60.72 | 1 | 6.90×10^-15^ |
|  |  | Model 3: Covariates+LS+PRS+LS×PRS | 0.001 | 8.01 | 6.56 | Model 3 vs 2 | 26.70 | 1 | 2.41×10^-07^ |
| Anxiety | LDI | Model 0: Covariates |  | 2.63 |  |  |  |  |  |
|  |  | Model 1: Covariates+LDI |  | 18.80 | 16.17 | Model 1 vs 0 | 8032.70 | 1 | < 2.20×10^-16^ |
|  |  | Model 2: Covariates+LDI+PRS | 0.1 | 19.10 | 16.47 | Model 2 vs 1 | 151.36 | 1 | < 2.20×10^-16^ |
|  |  | Model 3: Covariates+LDI+PRS+LDI×PRS | 0.1 | 19.19 | 16.56 | Model 3 vs 2 | 42.32 | 1 | 7.85×10^-11^ |
|  | LTE | Model 0: Covariates |  | 2.62 |  |  |  |  |  |
|  |  | Model 1: Covariates+LTE |  | 6.33 | 3.71 | Model 1 vs 0 | 1600.90 | 1 | < 2.20×10^-16^ |
|  |  | Model 2: Covariates+LTE+PRS | 0.1 | 6.86 | 4.24 | Model 2 vs 1 | 230.31 | 1 | < 2.20×10^-16^ |
|  |  | Model 3: Covariates+LTE+PRS+LTE×PRS | 0.1 | 6.90 | 4.29 | Model 3 vs 2 | 19.96 | 1 | 7.93×10^-06^ |
|  | SS | Model 0: Covariates |  | 2.63 |  |  |  |  |  |
|  |  | Model 1: Covariates+SS |  | 7.08 | 4.45 | Model 1 vs 0 | 1889.90 | 1 | < 2.20×10^-16^ |
|  |  | Model 2: Covariates+SS+PRS | 0.05 | 7.57 | 4.94 | Model 2 vs 1 | 209.75 | 1 | < 2.20×10^-16^ |
|  |  | Model 3: Covariates+SS+PRS+SS×PRS | 0.05 | 7.63 | 4.99 | Model 3 vs 2 | 24.51 | 1 | 7.42×10^-07^ |
|  | CT | Model 0: Covariates |  | 2.91 |  |  |  |  |  |
|  |  | Model 1: Covariates+CT |  | 9.32 | 6.41 | Model 1 vs 0 | 1412.50 | 1 | < 2.20×10^-16^ |
|  |  | Model 2: Covariates+CT+PRS | 0.05 | 9.78 | 6.86 | Model 2 vs 1 | 101.14 | 1 | < 2.20×10^-16^ |
|  |  | Model 3: Covariates+CT+PRS+CT×PRS | 0.05 | 9.78 | 6.87 | Model 3 vs 2 | 2.04 | 1 | 0.1536 |
|  | LS | Model 0: Covariates |  | 2.91 |  |  |  |  |  |
|  |  | Model 1: Covariates+LS |  | 10.19 | 7.28 | Model 1 vs 0 | 1620.60 | 1 | < 2.20×10^-16^ |
|  |  | Model 2: Covariates+LS+PRS | 0.05 | 10.68 | 7.77 | Model 2 vs 1 | 110.19 | 1 | < 2.20×10^-16^ |
|  |  | Model 3: Covariates+LS+PRS+LS×PRS | 0.05 | 10.77 | 7.86 | Model 3 vs 2 | 20.24 | 1 | 6.86×10^-06^ |

Abbreviations: PRS, polygenic risk score; LDI, Long-term difficulties inventory; LTE, List of threatening experiences; SS, Social support; CT, Childhood trauma; LS, Loneliness; PT: p-threshold for PRS. Covariates included age, sex, chip (CytoSNP or GSA) and 10 principal components.

**Table S6. The effects of PRS, stress and their interactions for depression and anxiety in total sample, adults and children**

| Variables | | | Total sample | | | | | Adults | | | | | Children | | | | |
| --- | --- | --- | --- | --- | --- | --- | --- | --- | --- | --- | --- | --- | --- | --- | --- | --- | --- |
|  |  |  | Best-fit PT | n | beta | p | R^2^ (%) | Best-fit PT | n | beta | p | R^2^ (%) | Best-fit PT | n | beta | p | R^2^ (%) |
| Dep | PRS | PRS_dep | 0.05 | 41,524 | 0.1105 | <2.00×10^-16^ | 0.66 | 0.05 | 38,660 | 0.1149 | <2.00×10^-16^ | 0.69 | 1×10^-4^ | 2,864 | 0.0403 | 0.03 | 0.21 |
|  | Stress | LDI | -- | 40,425 | 0.4195 | <2.00×10^-16^ | 9.54 | -- | 37,688 | 0.4350 | <2.00×10^-16^ | 10.19 | -- | 2,737 | 0.6235 | <2.00×10^-16^ | 7.59 |
|  |  | LTE | -- | 40,521 | 0.2504 | <2.00×10^-16^ | 3.38 | -- | 37,784 | 0.2382 | <2.00×10^-16^ | 3.25 | -- | 2,736 | 0.2649 | <2.00×10^-16^ | 6.22 |
|  |  | Social support | -- | 39,593 | -0.2572 | <2.00×10^-16^ | 3.30 | -- | 37,967 | -0.3052 | <2.00×10^-16^ | 3.69 | -- | 1,626 | -0.1206 | 9.89×10^-10^ | 3.86 |
|  | G×E | PRS_dep*LDI | 0.5 | 40,425 | 0.0541 | <2.00×10^-16^ | 0.17 | 0.5 | 37,688 | 0.0515 | 2.11×10^-15^ | 0.15 | 0.1 | 2,737 | 0.0862 | 0.0795 | 0.15 |
|  |  | PRS_dep*LTE | 1×10^-5^ | 40,521 | 0.0316 | 9.63×10^-07^ | 0.05 | 1×10^-5^ | 37,784 | 0.0324 | 1.63×10^-06^ | 0.06 | 0.1 | 2,736 | 0.0352 | 0.1102 | 0.12 |
|  |  | PRS_dep*social support | 0.5 | 39,593 | -0.0417 | 1.87×10^-10^ | 0.09 | 0.5 | 37,967 | -0.0423 | 4.42×10^-08^ | 0.07 | 1.0 | 1,626 | 0.0229 | 0.2844 | 0.12 |
| Anx | PRS | PRS_anx | 0.1 | 41,451 | 0.1808 | <2.00×10^-16^ | 0.64 | 0.1 | 38,586 | 0.1919 | <2.00×10^-16^ | 0.69 | 0.01 | 2,865 | 0.0534 | 0.0193 | 0.21 |
|  | Stress | LDI | -- | 40,354 | 0.8934 | <2.00×10^-16^ | 16.60 | -- | 37,616 | 0.8885 | <2.00×10^-16^ | 16.35 | -- | 2,738 | 0.7882 | <2.00×10^-16^ | 6.54 |
|  |  | LTE | -- | 40,442 | 0.4284 | <2.00×10^-16^ | 3.76 | -- | 37,704 | 0.4032 | <2.00×10^-16^ | 3.68 | -- | 2,738 | 0.3358 | <2.00×10^-16^ | 5.37 |
|  |  | Social support | -- | 39,520 | -0.4807 | <2.00×10^-16^ | 4.35 | -- | 37,894 | -0.5026 | <2.00×10^-16^ | 4.04 | -- | 1,626 | -0.1438 | 8.50×10^-11^ | 2.56 |
|  | G×E | PRS_anx*LDI | 0.1 | 40,354 | 0.0546 | 8.40×10^-09^ | 0.08 | 0.1 | 37,616 | 0.0526 | 1.26×10^-07^ | 0.07 | 5×10^-8^ | 2,738 | 0.1085 | 0.0556 | 0.12 |
|  |  | PRS_anx*LTE | 0.1 | 40,442 | 0.0385 | 2.10×10^-04^ | 0.05 | 0.1 | 37,704 | 0.0384 | 4.36×10^-04^ | 0.04 | 0.5 | 2,738 | 0.0499 | 0.0408 | 0.15 |
|  |  | PRS_anx*social support | 0.05 | 39,520 | -0.0452 | 1.46×10^-05^ | 0.05 | 0.05 | 37,894 | -0.0390 | 2.05×10^-03^ | 0.04 | 0.01 | 1,626 | -0.0270 | 0.2338 | 0.08 |

Abbreviations: PRS, polygenic risk score; LDI, Long-term difficulties inventory; LTE, List of threatening experiences; PT: p-threshold for PRS; R^2^: the variance explained by the predictor. All models adjusted for age, sex, chip (CytoSNP or GSA) and 10 principal components.

**Table S7. The effect of PRS, stress, and their interactions among total and cleaned depression phenotype**

|  | Variables | Total depression phenotype | | | | Cleaned depression phenotype | | | |
| --- | --- | --- | --- | --- | --- | --- | --- | --- | --- |
|  |  | n | beta | p | R^2^ (%) | n | beta | p | R^2^ (%) |
| PRS | PRS_dep_0.05 | 41,524 | 0.1105 | <2.00×10^-16^ | 0.66 | 32,772 | 0.1174 | <2.00×10^-16^ | 0.72 |
| Stress | LDI | 40,425 | 0.4195 | <2.00×10^-16^ | 9.54 | 31,939 | 0.4474 | <2.00×10^-16^ | 10.10 |
|  | LTE | 40,521 | 0.2504 | <2.00×10^-16^ | 3.38 | 32,014 | 0.2795 | <2.00×10^-16^ | 4.01 |
|  | Social support | 39,593 | -0.2572 | <2.00×10^-16^ | 3.30 | 31,395 | -0.2800 | <2.00×10^-16^ | 3.69 |
|  | Childhood trauma | 20,030 | 0.3191 | <2.00×10^-16^ | 5.91 | 15,424 | 0.3717 | <2.00×10^-16^ | 6.54 |
|  | Loneliness | 20,035 | 0.3376 | <2.00×10^-16^ | 6.57 | 15,423 | 0.3788 | <2.00×10^-16^ | 7.04 |
| G×E | PRS_dep_0.5*LDI | 40,425 | 0.0541 | <2.00×10^-16^ | 0.17 | 31,939 | 0.0806 | <2.00×10^-16^ | 0.31 |
|  | PRS_dep_1.0e-5*LTE | 40,521 | 0.0316 | 9.63×10^-07^ | 0.05 | 32,014 | 0.0406 | 1.68×10^-07^ | 0.08 |
|  | PRS_dep_0.5*social support | 39,593 | -0.0417 | 1.87×10^-10^ | 0.09 | 31,395 | -0.0565 | 3.42×10^-13^ | 0.15 |
|  | PRS_dep_0.5*childhood trauma | 20,030 | 0.0384 | 1.78×10^-05^ | 0.09 | 15,424 | 0.0635 | 1.47×10^-08^ | 0.20 |
|  | PRS_dep_0.001*loneliness | 20,035 | 0.0486 | 1.06×10^-07^ | 0.12 | 15,423 | 0.0632 | 1.18×10^-08^ | 0.19 |

Abbreviations: LDI, Long-term difficulties inventory; LTE, List of threatening experiences. All models adjusted for age, sex, chip (CytoSNP or GSA) and 10 principal components.

**Table S8. Correlations between PRSs and stress-related exposures**

| PRS | Stress-related exposure | Correlation | *p* |
| --- | --- | --- | --- |
| PRS_dep_0.5 | LDI | 0.08 | < 2.20×10^-16^ |
| PRS_dep_1.0e-5 | LTE | 0.03 | 8.49×10^-09^ |
| PRS_dep_0.5 | social support | -0.04 | < 2.20×10^-16^ |
| PRS_dep_0.5 | childhood trauma | 0.08 | < 2.20×10^-16^ |
| PRS_dep_0.001 | loneliness | 0.04 | 9.43×10^-08^ |
| PRS_anx_0.1 | LDI | 0.06 | < 2.20×10^-16^ |
| PRS_anx_0.1 | LTE | 0.04 | 1.31×10^-13^ |
| PRS_anx_0.05 | social support | -0.03 | 1.10×10^-09^ |
| PRS_anx_0.05 | childhood trauma | 0.07 | < 2.20×10^-16^ |
| PRS_anx_0.05 | loneliness | 0.05 | 9.90×10^-12^ |

Abbreviations: LDI, Long-term difficulties inventory; LTE, List of threatening experiences.

**Table S9. The effect of PRS, childhood trauma and subscales and their interactions for depression and anxiety**

| Phenotype | Stress |  | Best-fit PT | n | Beta | *p* | R^2^ (%) |
| --- | --- | --- | --- | --- | --- | --- | --- |
| Depression | Childhood trauma (CT) | CT |  | 20,030 | 0.3023 | < 2.20×10^-16^ | 5.43 |
|  |  | PRS | 0.5 |  | 0.0831 | < 2.20×10^-16^ | 0.38 |
|  |  | CT×PRS | 0.5 |  | 0.0384 | 1.78×10^-05^ | 0.09 |
|  | Emotional abuse (EA) | EA |  | 20,030 | 0.2930 | < 2.20×10^-16^ | 4.84 |
|  |  | PRS | 0.05 |  | 0.0875 | < 2.20×10^-16^ | 0.39 |
|  |  | EA×PRS | 0.05 |  | 0.0448 | 3.04×10^-06^ | 0.11 |
|  | Emotional neglect (EN) | EN |  | 20,024 | 0.1506 | < 2.20×10^-16^ | 1.34 |
|  |  | PRS | 0.01 |  | 0.0983 | < 2.20×10^-16^ | 0.50 |
|  |  | EN×PRS | 0.01 |  | 0.0438 | 3.17×10^-06^ | 0.11 |
|  | Physical abuse (PA) | PA |  | 20,029 | 0.1828 | < 2.20×10^-16^ | 1.78 |
|  |  | PRS | 1×10^-05^ |  | 0.0595 | 4.09×10^-10^ | 0.21 |
|  |  | PA×PRS | 1×10^-05^ |  | 0.0194 | 0.04 | 0.02 |
|  | Physical neglect (PN) | PN |  | 20,036 | 0.1811 | < 2.20×10^-16^ | 1.80 |
|  |  | PRS | 1.0 |  | 0.0988 | < 2.20×10^-16^ | 0.52 |
|  |  | PN×PRS | 1.0 |  | 0.0484 | 7.09×10^-07^ | 0.11 |
|  | Sexual abuse (SA) | SA |  | 20,022 | 0.2193 | < 2.20×10^-16^ | 2.41 |
|  |  | PRS | 0.0001 |  | 0.0722 | 1.62×10^-14^ | 0.27 |
|  |  | SA×PRS | 0.0001 |  | -0.0183 | 0.06 | 0.02 |
| Anxiety | Childhood trauma (CT) | CT |  | 20,010 | 0.5486 | < 2.20×10^-16^ | 6.41 |
|  |  | PRS | 0.05 |  | 0.1583 | < 2.20×10^-16^ | 0.46 |
|  |  | CT×PRS | 0.05 |  | 0.0192 | 0.2009 | 0.01 |
|  | Emotional abuse (EA) | EA |  | 20,010 | 0.5411 | < 2.20×10^-16^ | 5.68 |
|  |  | PRS | 0.0001 |  | 0.0514 | 8.78×10^-04^ | 0.05 |
|  |  | EA×PRS | 0.0001 |  | 0.0182 | 0.26 | 0.01 |
|  | Emotional neglect (EN) | EN |  | 20,004 | 0.3291 | < 2.20×10^-16^ | 2.31 |
|  |  | PRS | 0.05 |  | 0.1810 | < 2.20×10^-16^ | 0.60 |
|  |  | EN×PRS | 0.05 |  | 0.0235 | 0.13 | 0.01 |
|  | Physical abuse (PA) | PA |  | 20,009 | 0.2848 | < 2.20×10^-16^ | 1.54 |
|  |  | PRS | 0.001 |  | 0.1248 | 1.55×10^-15^ | 0.29 |
|  |  | PA×PRS | 0.001 |  | -0.0387 | 0.02 | 0.02 |
|  | Physical neglect (PN) | PN |  | 20,016 | 0.3166 | < 2.20×10^-16^ | 1.90 |
|  |  | PRS | 0.001 |  | 0.1279 | 5.55×10^-16^ | 0.30 |
|  |  | PN×PRS | 0.001 |  | 0.0258 | 0.11 | 0.02 |
|  | Sexual abuse (SA) | SA |  | 20,002 | 0.3731 | < 2.20×10^-16^ | 2.47 |
|  |  | PRS | 1×10^-05^ |  | 0.0702 | 1.41×10^-05^ | 0.10 |
|  |  | SA×PRS | 1×10^-05^ |  | 0.0419 | 0.01 | 0.02 |

All models adjusted for age, sex, chip (CytoSNP or GSA) and 10 principal components.

**Part 2: Supplementary Methods**

**Measurement**

**(1) Depression and Anxiety**

For adults, current depression and anxiety were measured using the Mini-international Neuropsychiatric interview (MINI) ^[1]^. The MINI is a brief structured interview for diagnosing psychiatric disorders as defined by the fourth edition of the Diagnostic and Statistical Manual of Mental Disorders (DSM-IV) and the International Classification of Diseases (ICD-10) ^[1]^. For those stress-related exposures measured at baseline (such as LDI, LTE and social support), we calculated sum score of current depression and anxiety using the MINI without skips at baseline, for the MINI with skips at baseline (n=19,992), we used the MINI without skips at follow-up to capture continuous depression and anxiety. As childhood trauma and loneliness were only measured at follow-up (n=20,152), we preferentially used sum scores of depression and anxiety measured at follow-up as outcome (n=18,635) supplemented by sum scores of current depression and anxiety at baseline for participants who did not have measurements of depression and anxiety at follow-up (n=1,517). (Items are in the Table S10)

**Table S10. Items for calculating sum scores of depression and anxiety in MINI**

| Phenotypes | Items | Questions | Answers |
| --- | --- | --- | --- |
| Sum score of depression for adults | A1 | Have you been consistently depressed or down, most of the day, nearly every day, for the past two weeks? | 0=no  1=yes |
|  | A2 | In the past two weeks, have you been much less interested in most things or much less able to enjoy the things you used to enjoy most of the time? |  |
|  | A3A | Was your appetite decreased or increased nearly every day? Did your weight decrease or increase without trying intentionally? |  |
|  | A3B | Did you have trouble sleeping nearly every night (difficulty falling asleep, waking up in the middle of the night, early morning wakening or sleeping excessively)? |  |
|  | A3C | Did you talk or move more slowly than normal or were you fidgety, restless or having trouble sitting still almost every day? |  |
|  | A3D | Did you feel tired or without energy almost every day? |  |
|  | A3E | Did you feel worthless or guilty almost every day? |  |
|  | A3F | Did you have difficulty concentrating or making decisions almost every day? |  |
|  | A3G | Did you repeatedly consider hurting yourself, feel suicidal, or wish that you were dead? |  |
|  | B1 | Have you felt sad, low or depressed most of the time for the last two years? |  |
| Sum score of anxiety for adults | O1A | Have you worried excessively or been anxious about several problems of daily life (problems at work, at home or in your close circle) over the past 6 months? | 0=no  1=yes |
|  | O3A | When you were anxious over the past 6 months, did you, most of the time, feel restless, keyed up or on edge? |  |
|  | O3B | When you were anxious over the past 6 months, did you, most of the time, feel tense? |  |
|  | O3C | When you were anxious over the past 6 months, did you, most of the time, feel tired, weak or exhausted easily? |  |
|  | O3D | When you were anxious over the past 6 months, did you, most of the time, have difficulty concentrating or find your mind going blank? |  |
|  | O3E | When you were anxious over the past 6 months, did you, most of the time, feel irritable? |  |
|  | O3F | When you were anxious over the past 6 months, did you, most of the time, have difficulty sleeping (difficulty falling asleep, waking up in the middle of the night, early morning wakening or sleeping excessively)? |  |
|  | E1 | Have you, on more than one occasion, had spells or attacks when you suddenly felt anxious, frightened, uncomfortable or uneasy, even in situations where most people would not feel that way? |  |
|  | F1 | Do you feel anxious or uneasy in places or situations where you might have a panic attack or the panic-like symptoms we just spoke about, or where help might not be available or escape might be difficult: like being in a crowd, standing in a line (queue), when you are alone away from home or alone at home, or when crossing a bridge, traveling in a bus, train or car? |  |
|  | G1 | In the past month, were you fearful or embarrassed being watched, being the focus of attention, or fearful of being humiliated? This includes things like speaking in public, eating in public or with others, writing while someone watches, or being in social situations. |  |

For children, depression (n=2,864) and anxiety (n=2,865) were measured using children’s behavior questionnaires at baseline, combining the Child Behavior Checklist (CBCL) ^[2]^ for ages 8-17 years and the Youth Self-Report (YSR) ^[3]^ for 13-17 years. Sum scores of depression and anxiety were calculated as continuous traits (Items are in the Table S11).

**Table S11. Items for calculating sum scores of depression and anxiety in CBCL and YSR**

| Phenotypes | Age | Items | Questions | Answers |
| --- | --- | --- | --- | --- |
| Sum score of depression for children | Parent-report CBCL  (8-17 years) | CBCL5 | There are not many things it likes | 0=not at all  1=a little bit or sometimes  2=clearly or often |
|  |  | CBCL14 | Cries a lot |  |
|  |  | CBCL18 | Self-harms, or tries to commit suicide |  |
|  |  | CBCL24 | Does not eat well |  |
|  |  | CBCL35 | Feels useless or inferior |  |
|  |  | CBCL52 | Feels very guilty |  |
|  |  | CBCL54 | Is very tired without reason |  |
|  |  | CBCL76 | Sleeps less than most boys and girls |  |
|  |  | CBCL77 | Sleeps more than most boys and girls |  |
|  |  | CBCL91 | Talks about wanting to kill itself |  |
|  |  | CBCL100 | Problems sleeping |  |
|  |  | CBCL102 | Is not very active, moves slowly or has little energy |  |
|  |  | CBCL103 | Is unhappy, sad or depressed |  |
|  | Self-report YSR  (13-17 years) | ACHYSR5 | There is little that I like |  |
|  |  | ACHYSR14 | I cry a lot |  |
|  |  | ACHYSR18 | I try to deliberately hurt or kill myself |  |
|  |  | ACHYSR24 | I do not eat as well as I should |  |
|  |  | ACHYSR35 | I feel useless or inferior |  |
|  |  | ACHYSR52 | I feel very guilty |  |
|  |  | ACHYSR54 | I feel very tired without knowing why |  |
|  |  | ACHYSR83 | I sleep less than most boys and girls |  |
|  |  | ACHYSR84 | I sleep more than most boys and girls |  |
|  |  | ACHYSR98 | I think about killing myself |  |
|  |  | ACHYSR107 | I have trouble sleeping |  |
|  |  | ACHYSR109 | I don't have a lot of energy |  |
|  |  | ACHYSR110 | I am unhappy, sad or depressed |  |
| Sum score of anxiety for children | Parent-report CBCL  (8-17 years) | CBCL11 | Clings to adults or is too dependent | 0=not at all  1=a little bit or sometimes  2=clearly or often. |
|  |  | CBCL29 | Is afraid of certain animals, situations or locations other than school |  |
|  |  | CBCL30 | Is afraid to go to school |  |
|  |  | CBCL45 | Is nervous, twitchy or tense |  |
|  |  | CBCL50 | Is overly scared or anxious |  |
|  |  | CBCL112 | Worries |  |
|  | Self-report YSR  (13-17 years) | ACHYSR11 | I am too dependent on adults |  |
|  |  | ACHYSR29 | I am afraid of certain animals, situations or locations other than school |  |
|  |  | ACHYSR30 | I am afraid to go to school |  |
|  |  | ACHYSR45 | I am nervous, highly-strung or tense |  |
|  |  | ACHYSR50 | I am too frightened or scared |  |
|  |  | ACHYSR119 | I worry a lot |  |

**(2) Long-term Difficulties Inventory (LDI)**

Long-term difficulties in the past year of adults were measured at baseline using the Long-term Difficulties Inventory (LDI) ^[4]^. Items are in the Table S12.

**Table S12. Items for calculating sum scores of LDI for adults at baseline**

| Phenotypes | Items | Questions | Answers |
| --- | --- | --- | --- |
| Sum score of LDI for adults |  | To what extent did you experience difficulties and stress related to this aspect of your life? | 0=not 1=somewhat 2=much |
|  | GLLM1 | Home and living (e.g. accommodation too small, could not find a home, noise) |  |
|  | GLLM2 | At or with work (e.g. too demanding, conflicts with boss, (imminent) dismissal) |  |
|  | GLLM3 | Relationship with friends or acquaintances (e.g. quarrels, lack of support) |  |
|  | GLLM4 | Relationship with your partner (e.g. jealousy, conflicts, doubt about the relationship, quarrels) |  |
|  | GLLM5 | Relationship with your children (e.g. frequent conflicts, lack of respect for you) |  |
|  | GLLM6 | Relationship with your parents (e.g. frequent conflicts, lack of acceptance) |  |
|  | GLLM7 | Relationship with other relatives (e.g. conflicts, lack of acceptance) |  |
|  | GLLM8 | Free time (e.g. too little or too much free time) |  |
|  | GLLM9 | Finances (e.g. major debts, insufficient income) |  |
|  | GLLM10 | Your health (e.g. regularly ill, longer-term disorders) |  |
|  | GLLM11 | School/study (too difficult, cannot be combined with other tasks) |  |
|  | GLLM12 | Faith, church or religion (e.g. doubt, conflicts with your minister) |  |

For children, long-term difficulties in the past 2 years were measured at baseline by parent-reports 13 items of influence of stress events for children (n=2,738) ^[5]^. Items are in the Table S13.

**Table S13. Items for calculating sum scores of LDI for children at baseline**

| Phenotypes | Items | Questions | Answers |
| --- | --- | --- | --- |
| Parent-report LDI for children |  | How much impact does that situation in the past 2 years have on your child right now? | 0=none 1=a bit 2=quite a lot 3=very much |
|  | chstress1b | Your child has a chronic disease or disability |  |
|  | chstress2b | Another family member has a chronic disease or disability |  |
|  | chstress3b | Your child is experiencing a very high workload at school |  |
|  | chstress4b | There are problems related to your home (too small, too much noise, too busy) |  |
|  | chstress5b | There are problems in the neighbourhood (eg vandalism, not safe) |  |
|  | chstress6b | A family member is/was at home for longer than 3 months because of unemployment or incapacity for work |  |
|  | chstress7b | Your family has financial problems |  |
|  | chstress8b | Your child has fewer friends than it would like |  |
|  | chstress9b | Your child is being bullied at school or in the street |  |
|  | chstress10b | Your child does not get along with a member of the family |  |
|  | chstress11b | Your child does not get along with someone else |  |
|  | chstress12b | Other family members do not get along |  |
|  | chstress13b | You and your partner are divorced (and are no longer living together) |  |

**(3) List of Threatening Events (LTE)**

Stressful life events in the past year of adults were assessed at baseline using the List of Threatening Events (LTE) ^[4]^. Items are in the Table S14.

**Table S14. Items for calculating sum scores of LTE for adults at baseline**

| Phenotypes | Items | Questions | Answers |
| --- | --- | --- | --- |
| Sum score of LTE for adults |  | Could you indicate whether you experienced this unpleasant event in the past year? | 0=no  1=yes |
|  | BRUGLA1 | You were severely ill, severely injured or a victim of violence |  |
|  | BRUGLA2 | A relative was severely ill, severely injured or a victim of violence |  |
|  | BRUGLA3 | A parent, child, brother, sister or partner died |  |
|  | BRUGLA4 | A good friend or close relative died |  |
|  | BRUGLA5 | You and your partner split up |  |
|  | BRUGLA6 | You ended a long-term relationship with a good friend or relative |  |
|  | BRUGLA7 | You got into a serious problem with a good friend, relative or neighbour |  |
|  | BRUGLA8 | You lost your job and haven't been able to find work again |  |
|  | BRUGLA9 | You were fired |  |
|  | BRUGLA10 | You faced severe financial difficulties |  |
|  | BRUGLA11 | You got into trouble with the police or the law |  |
|  | BRUGLA12 | You lost money or valuables through theft or loss |  |

For children, stressful life events in the past 2 years were measured at baseline by parent-report questionnaires with 13 items relevant to whether the stressful event occurred in the past 2 years (n=2,738) ^[5]^. Items are in the Table S15.

**Table S15. Items for calculating sum scores of LTE for children at baseline**

| Phenotypes | Items | Questions | Answers |
| --- | --- | --- | --- |
| Parent-report LTE for children |  | Did this situation apply to your child in the past 2 years? | 0=no  1=yes |
|  | chstress1 | Your child has a chronic disease or disability |  |
|  | chstress2 | Another family member has a chronic disease or disability |  |
|  | chstress3 | Your child is experiencing a very high workload at school |  |
|  | chstress4 | There are problems related to your home (too small, too much noise, too busy) |  |
|  | chstress5 | There are problems in the neighbourhood (eg vandalism, not safe) |  |
|  | chstress6 | A family member is/was at home for longer than 3 months because of unemployment or incapacity for work |  |
|  | chstress7 | Your family has financial problems |  |
|  | chstress8 | Your child has fewer friends than it would like |  |
|  | chstress9 | Your child is being bullied at school or in the street |  |
|  | chstress10 | Your child does not get along with a member of the family |  |
|  | chstress11 | Your child does not get along with someone else |  |
|  | chstress12 | Other family members do not get along |  |
|  | chstress13 | You and your partner are divorced (and are no longer living together) |  |

**(4) Social support**

Social support in the past year of adults was assessed at baseline using the 9 items short version of the Social Production Function Instrument for the Level of well-being (SPF-IL) ^[6]^. Items are in the Table S16.

**Table S16. Items for calculating sum scores of social support for adults at baseline**

| Phenotypes | Items | Questions | Answers |
| --- | --- | --- | --- |
| Social support |  | The following questions are about how you felt in the past year | 0=never 1=sometimes 2=often 3=always |
|  | SPF-IL1 | Do people pay attention to you? |  |
|  | SPF-IL2 | Do people help you if you have a problem? |  |
|  | SPF-IL3 | Do you feel that people really love you? |  |
|  | SPF-IL4 | There are situations in which we deal with groups of people, for example at home, at work or during our leisure time. do others appreciate your role in the group? |  |
|  | SPF-IL5 | When you are at school, at work, with family, at an association or in church, do you feel like you belong? |  |
|  | SPF-IL6 | Do others appreciate the things you do? |  |
|  | SPF-IL7 | Do people think you do better than others? |  |
|  | SPF-IL8 | Do people find you an influential person? |  |
|  | SPF-IL9 | Are you known for the things you have accomplished? |  |

For children, social support in the past 7 days was measured at follow-up combining 8-item self-report and parent-report PROMIS-29 Profile (n=1,626) ^[7]^. Items are in the Table S17.

**Table S17. Items for calculating sum scores of social support for children at follow-up**

| Phenotypes | Items | Questions (in the past 7 days) | Answers |
| --- | --- | --- | --- |
| Parent-report social support for children (4-17 years) | 1 | My child felt accepted by other children of the same age | 0=never 1=almost never 2=sometimes 3=often 4=almost always |
|  | 2 | My child could count on their friends |  |
|  | 3 | My child was able to make friends easily |  |
|  | 4 | My child and their friends helped each other |  |
|  | 5 | Other children wanted to be my child’s friend |  |
|  | 6 | Other children wanted to be with my child |  |
|  | 7 | Other children wanted to talk to my child |  |
|  | 8 | Other children wanted to play with my child |  |
| Self-report social support for children (13-17 years) | 1 | I felt accepted by other children of my age | 0=never 1=almost never 2=sometimes 3=often 4=almost always |
|  | 2 | I was able to rely on my friends |  |
|  | 3 | I was able to make friends easily |  |
|  | 4 | My friends and I help each other |  |
|  | 5 | Other children wanted to be my friend |  |
|  | 6 | Other children wanted to be with me |  |
|  | 7 | Other children wanted to talk to me |  |
|  | 8 | I could talk to my friends about anything |  |

**(4) Childhood trauma**

Childhood trauma was measured among adults 2 years after the second assessment, using a 28-item retrospective self-report Childhood Trauma Questionnaire-Short Form (CTQ-SF) ^[8]^. There are 5 items on each clinical scale of the CTQ-SF plus an additional 3-item minimization/denial scale. Items are in the Table S18.

**Table S18. Items for calculating sum scores of childhood trauma for adults by second assessment follow-up questionnaire**

| Phenotypes | Items | Questions | Answers |
| --- | --- | --- | --- |
| Emotional Abuse | CTQ3 | During my childhood family members called me ‘stupid’, ‘lazy’ or ‘ugly’ | 1=never true 2=rarely true 3=sometimes true 4=often true 5=very often true |
|  | CTQ8 | During my childhood I felt my parents wished I had never been born. |  |
|  | CTQ14 | During my childhood people in my family said hurtful or offensive things to me. |  |
|  | CTQ18 | During my childhood I felt someone in my family hated me. |  |
|  | CTQ25 | During my childhood I believe I was abused emotionally. |  |
| Physical  Abuse | CTQ09 | During my childhood I was hit by a family member so hard that I had to go to the doctor or hospital. | 1=never true 2=rarely true 3=sometimes true 4=often true 5=very often true |
|  | CTQ11 | During my childhood I was hit so hard by family members that I was left with bruises or scars. |  |
|  | CTQ12 | During my childhood I was punished with a belt, board, rope or other hard object. |  |
|  | CTQ15 | During my childhood I believe I was abused physically. |  |
|  | CTQ17 | During my childhood I was hit so hard that it was noticed by e.g. a teacher, neighbour or doctor. |  |
| Sexual Abuse | CTQ20 | During my childhood someone tried to touch me sexually or get me to touch them. | 1=never true 2=rarely true 3=sometimes true 4=often true 5=very often true |
|  | CTQ21 | During my childhood someone threatened to hurt me or tell lies about me if I refused to engage in a sexual act with him or her. |  |
|  | CTQ23 | During my childhood someone wanted me to do sexual things or let me watch sexual things. |  |
|  | CTQ24 | During my childhood I was molested by someone. |  |
|  | CTQ27 | During my childhood I believe I was abused sexually. |  |
| Emotional Neglect | CTQ5 | During my childhood there was someone in my family who made me feel important and special. | 5=never true 4=rarely true 3=sometimes true 2=often true 1=very often true |
|  | CTQ7 | During my childhood I felt I was loved. |  |
|  | CTQ13 | During my childhood family members stuck together. |  |
|  | CTQ19 | During my childhood the members of my family felt connected. |  |
|  | CTQ28 | During my childhood my family was a source of strength and support. |  |
| Physical Neglect | CTQ1 | During my childhood I did not have enough to eat. | 1=never true 2=rarely true 3=sometimes true 4=often true 5=very often true |
|  | CTQ4 | During my childhood my parents were too drunk or stoned (under the influence of drugs) to care for the family. |  |
|  | CTQ6 | During my childhood I had to wear dirty clothes. |  |
|  | CTQ2 | During my childhood I knew there was someone to care for me and protect me. | 5=never true 4=rarely true 3=sometimes true 2=often true 1=very often true |
|  | CTQ26 | During my childhood there was someone to take me to the doctor if necessary. |  |
| Minimization | CTQ10 | During my childhood there was nothing I wanted to change about my family. | 1=very often true 0=others |
|  | CTQ16 | During my childhood I had a perfect childhood. |  |
|  | CTQ22 | During my childhood I grew up in the best possible family. |  |

**(5) Loneliness**

Loneliness was measured using the 6-item De Jong Gierveld Loneliness Scale ^[9]^ among adults 2 years after the second assessment. Items are in the Table S19.

**Table S19. Items for calculating sum scores of loneliness for adults by second assessment follow-up questionnaire**

| Phenotypes | Items | Questions | Answers |
| --- | --- | --- | --- |
| Loneliness | 1 | I experience an emptiness around me | 5=yes! 4=yes 3=more or less 2=no 1=no! |
|  | 2 | I miss people around me |  |
|  | 3 | I often feel abandoned |  |
|  | 4 | I have enough people to fall back on in case of adversity | 1=yes! 2=yes 3=more or less 4=no 5=no! |
|  | 5 | I have many people that I can fully trust |  |
|  | 6 | there are enough people that I feel closely connected to |  |

**REFERENCES**

1. Sheehan DV, Lecrubier Y, Sheehan KH, Amorim P, Janavs J, Weiller E, et al. The Mini-International Neuropsychiatric Interview (M.I.N.I.): the development and validation of a structured diagnostic psychiatric interview for DSM-IV and ICD-10. *J Clin Psychiatry* 1998;59:22-33.
2. Achenbach TM. Manual for the Child Behavior Checklist/4-18 and 1991 Profiles. (Burlington: University of Vermont; 1991).
3. Achenbach TM. Manual for the Youth Self-Report and 1991 Profiles. (Burlington: University of Vermont; 1991).
4. Rosmalen JG, Bos EH, de Jonge P. Validation of the Long-term Difficulties Inventory (LDI) and the List of Threatening Experiences (LTE) as measures of stress in epidemiological population-based cohort studies. *Psychol Med* 2012;42:2599-2608.
5. Lifelines wiki. http://wiki-lifelines.web.rug.nl/doku.php?id=stress_general. 13 Apr, 2021.
6. Nieboer A, Lindenberg S, Boomsma A, Bruggen ACV. Dimensions Of Well-Being And Their Measurement: The Spf-Il Scale. *Soc Indic Res* 2005;73:313–353.
7. Lifelines wiki. http://wiki-lifelines.web.rug.nl/doku.php?id=general_health_promis. 13 Apr, 2021.
8. Thombs BD, Bernstein DP, Lobbestael J, Arntz A. A validation study of the Dutch Childhood Trauma Questionnaire-Short Form: factor structure, reliability, and known-groups validity. *Child Abuse Negl* 2009;33:518-523.
9. Gierveld JDJ, Tilburg TV. A shortened scale for overall, emotional and social loneliness. *Tijdschr Gerontol Geriatr* 2008;39:4-15.

**UMCG Genetics Lifelines Initiative (UGLI) group author**

LifeLines Cohort Study

Raul Aguirre-Gamboa (1), Patrick Deelen (1), Lude Franke (1), Jan A Kuivenhoven (2), Esteban A Lopera Maya (1), Ilja M Nolte (3), Serena Sanna (1), Harold Snieder (3), Morris A Swertz (1), Peter M. Visscher (3,4), Judith M Vonk (3), Cisca Wijmenga (1)

1. Department of Genetics, University of Groningen, University Medical Center Groningen, The Netherlands
2. Department of Pediatrics, University of Groningen, University Medical Center Groningen, The Netherlands
3. Department of Epidemiology, University of Groningen, University Medical Center Groningen, The Netherlands
4. Institute for Molecular Bioscience, The University of Queensland, Brisbane, Queensland, Australia.

**Acknowledgements**

Lifelines Cohort Study

The Lifelines Biobank initiative has been made possible by funding from the Dutch Ministry of Health, Welfare and Sport, the Dutch Ministry of Economic Affairs, the University Medical Center Groningen (UMCG the Netherlands), University of Groningen and the Northern Provinces of the Netherlands. The generation and management of GWAS genotype data for the Lifelines Cohort Study is supported by the UMCG Genetics Lifelines Initiative (UGLI). UGLI is partly supported by a Spinoza Grant from NWO, awarded to Cisca Wijmenga. The authors wish to acknowledge the services of the Lifelines Cohort Study, the contributing research centers delivering data to Lifelines, and all the study participants.
